# Renewable and Functional Latexes Synthesized by Polymerization-Induced Self-Assembly for UV-Curable Films

**DOI:** 10.1021/acsami.3c11657

**Published:** 2023-11-06

**Authors:** Jules Stouten, Huixing Cao, Andrij Pich, Katrien V. Bernaerts

**Affiliations:** † Aachen-Maastricht Institute for Biobased Materials (AMIBM), Faculty of Science and Engineering, Maastricht University, Brightlands Chemelot Campus, Urmonderbaan 22, 6167 RD Geleen, The Netherlands; ‡ DWI Leibniz-Institute for Interactive Materials, Aachen 52056, Germany; § Institute of Technical and Macromolecular Chemistry (ITMC), RWTH Aachen University, Aachen 52074, Germany

**Keywords:** composite, films, latex, renewable resources, sustainable chemistry, UV cross-linking

## Abstract

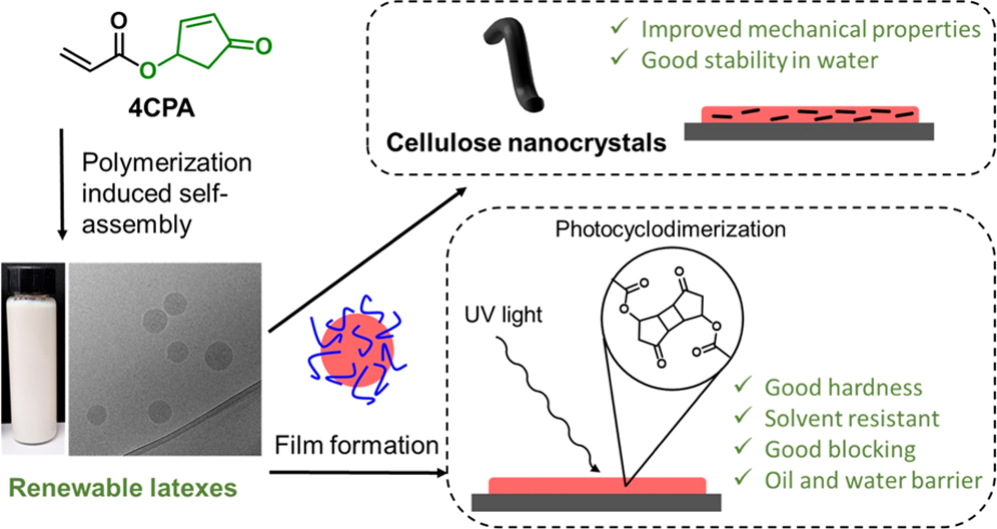

After the development of polymer coatings and films based on renewable resources, there remains a challenge of combining the advantages of water-borne acrylic latexes with the excellent physical properties of cross-linked solvent-borne coatings. After polymerization, the renewable 4-oxocyclopentenyl acrylate (4CPA) is capable of undergoing photocyclodimerization under UV light, yielding a cross-linked polyacrylate. In this work, we investigate the polymerization-induced self-assembly (PISA) of 4CPA with several renewable acrylic monomers in the presence of a macro-RAFT agent. The produced latexes have a small particle size, good colloidal stability, and are free of volatile organic compounds. After film formation and UV curing, flexible to rigid films can be obtained depending on the monomer composition and UV irradiation time. The cross-linked films show promise as oil and water barriers in paper coating applications. This work outlines the development and application of renewable and functional cross-linkable latexes synthesized by PISA.

## Introduction

Water-borne acrylic coatings play an important role in the modern world. They contribute to the durability, performance, and beautification of material surfaces in, for example, construction and (food) packaging. The drive to reduce hazardous volatile organic compound (VOC) levels in coating formulations led to the development and increased use of water-borne acrylic latex binders.^1^ These latex formulations require much less VOC to achieve film formation but suffer from certain downsides compared to solvent-based coatings, such as reduced barrier and mechanical properties, gloss, blocking, and chemical resistance.^2^ These downsides are linked to the latex particle coalescence mechanism and the absence of a cross-linked network after film formation.^3^ Another problem is that the acrylate esters used for latex binder synthesis are derived from fossil resources, the global production of which exceeds 5.2 million metric tons per year.^4^ Therefore, with the increasing global trend to reduce fossil resource consumption, it is evident that renewable alternatives need to be investigated to continue materials production.

Currently, biobased polymers are attracting increased attention; however, the polymerization of biobased acrylic monomers in industrially relevant aqueous emulsion processes remains considerably underexplored.^5^ This situation is partially due to the stricter conditions under which water-borne polymerization is executed, in contrast to solvent-borne polymerization, reducing the range of suitable monomers. In addition, the availability of biobased monomers containing radical polymerizable groups is limited. Therefore, the introduction of novel structures in polymer systems by functionalization of biobased building blocks with polymerizable acrylic groups is an important topic, moving toward the reduction of fossil components in latex systems.

The invention of controlled radical polymerization techniques has widened the scope of radical polymerization research; however, the translation of controlled radical polymerization performed in solution to an aqueous system is not straightforward. This complication has been alleviated by the development of a Polymerization-Induced Self-Assembly (PISA) procedure under Reversible Addition–Fragmentation chain-Transfer (RAFT) control.^6^ PISA requires the synthesis of a hydrophilic prepolymer, functionalized with a RAFT agent end-group, a macro-RAFT agent. Upon propagation from the macro-RAFT agent with hydrophobic monomers in the aqueous phase, spontaneous self-assembly into micelles with a core–shell structure takes place.^7^ The resulting particles are stabilized by a covalently bonded macro-RAFT agent. The RAFT-mediated PISA technique has also been employed to produce high solid content latexes, but its use for coatings has been reported only sparsely.^8−15^ Other benefits of producing polymers under RAFT control are that the molecular weight can be controlled and backbiting reactions are suppressed, reducing the amount of branching.^16,17^ These characteristics allow for the incorporation of biobased monomers with a more complex structure that adds functionality to the polymer backbone, limiting extensive cross-linking or side reactions.

In contrast to fossil-derived molecules, which consist mainly of aliphatic and aromatic hydrocarbons, biobased platform molecules typically contain a higher functional group density. This attribute requires not only a change in approach toward monomer and polymer synthesis but also affects the resulting polymer properties. Through various chemical conversions, it is possible to mimic conventional plastics by synthesizing biobased replacements for fossil-derived monomers that are chemically the same but consist of renewable carbon, for example, bio-PE. Alternatively, novel building blocks can be engineered to bring unique polymer properties or in such a way that they match the performance of conventional plastics. In this work, we utilize the high functional group density of a novel biobased acrylic monomer for use in UV-curable water-borne coatings. 4-Hydroxycyclopentenone (4HCP), which is obtained via the Piancatelli rearrangement of furfuryl alcohol,^18^ is modified with an acrylate group by esterification of the hydroxyl group. This reaction yields the radically polymerizable monomer 4-oxocyclopentenyl acrylate (4CPA). Polymerization of 4CPA via RAFT polymerization in solvents (not environmentally friendly) was recently reported,^17,19^ but in this work, emulsion polymerization will be explored. This is completely new and an important step to make the polymerization process environmentally friendly. The pendent cyclopentenone units on the resulting polymer can engage in a [2 + 2] photocyclodimerization under UV light to obtain polymeric networks.

**Figure 1:**
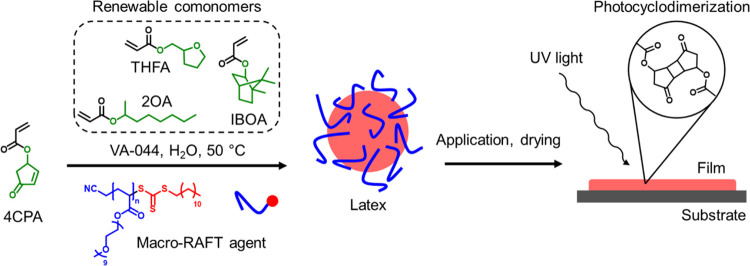
Schematic overview of the production of the latexes and their implementation as UV-cross-linked films.

Herein, the postpolymerization modification is utilized in emulsion polymerization with biobased comonomers to produce UV-curable latexes that are fully biobased. UV postcuring overcomes some of the aforementioned downsides related to thermoplastic latexes and results in films with good hardness, mechanical rigidity, chemical resistance, and blocking resistance. In addition, postcuring can offer proper film formation and increase in *T*_g_ after drying with lower amounts or no cosolvent/coalescing agent, thus lower levels of VOC. Previous reports on renewable UV-curable polyacrylate latexes required the addition of a cross-linker and initiator or lacked high solid content.^20,21^ In this work, we propose the use of a poly(oligo(ethylene glycol)) (POEGA) macro-RAFT agent for the RAFT-mediated PISA of renewable monomers 4CPA, tetrahydrofurfuryl acrylate (THFA), 2-octyl acrylate (2OA), and isobornyl acrylate (IBOA) (Figure 1). PISA avoids the need for free surfactants during emulsion polymerization, and as such, disadvantages of surfactants like leaching out, discoloration, or formation of snail trails in latex-based coatings can be circumvented. Snail trail refers to streaks or discolorations caused by the migration of water-soluble substances to the surface of the paint and their downward flow on freshly painted surfaces.^22^

By modification of the comonomer composition while maintaining the 4CPA feed, the polymer *T*_g_ and coating properties can be altered. Similarly, the UV curing time can be adjusted in order to tune the degree of cross-linking, hence affecting the final film properties. As a potential application, the UV-curable latex films were evaluated as water and oil barrier coatings for paper. The hydrophilic POEGA shell of the latex particles also improves the interaction and compatibility of the polymer with hydrophilic composite materials. In this way, we investigate the reinforcement with cellulose nanocrystals (CNC) to improve the mechanical properties of the resulting films.

## Experimental Section

### Materials

Azobis(isobutyronitrile) (AIBN, Sigma-Aldrich) was recrystallized from methanol prior to use. Isobornyl acrylate (IBOA, technical, Sigma-Aldrich), tetrahydrofurfuryl acrylate (THFA, 98%, abcr), 2-octyl acrylate (2OA, >98%, abcr), oligo(ethylene glycol) methyl ether acrylate (OEGA, average *M*_n_ of 480 g/mol, Sigma-Aldrich), and butyl acrylate (BA, ≥99%, Sigma-Aldrich) were passed over an alumina column and stored at −20 °C. 4-Oxocyclopent-2-ene-1-yl acrylate (4CPA) was synthesized according to a procedure mentioned in the literature and stored at −20 °C.^17^ Cyanomethyl dodecyl trithiocarbonate (CDT) was synthesized according to a procedure mentioned in the literature.^23^ 1,3,5-Trioxane (≥99%, Sigma-Aldrich), castor oil (Sigma-Aldrich), cellulose nanocrystals (CNC, length: 300–900 nm, width: 10–20 nm, Nanografi), naphthalene (>99%, Alfa-Aesar), 2,2′-Azobis[2-(2-imidazolin-2-yl)propane]dihydrochloride (VA-044, >98.0%, TCI), and CDCl_3_ (99.8%, Cambridge Isotope Laboratories) were used as received. All other solvents were obtained from Biosolve and used as received. The commercially available CHP204 (CH-polymers) is a noncurable styrene-acrylate polymer latex with a *T*_g_ of 10 °C and was used as a reference. The latex with a solid content of 50 wt % was diluted to 40 wt % using deionized water.

### Determination of Reactivity Ratios

The reactivity ratios of 4CPA with IBOA, THFA, and 2OA were determined via the Jaacks’ method.^24^ To determine the reactivity ratios of one monomer pair, two polymerization reactions were executed with one monomer in large excess (95/5) relative to the other in each of the reactions. In an exemplary reaction, a 25 mL Schlenk flask equipped with a magnetic stirrer was charged with 4CPA (500 mg, 3.29 mmol, 50 equiv), 2OA (30 mg, 0.16 mmol, 2.5 equiv), AIBN (1 mg, 6.56 μmol, 0.1 equiv), CDT (21 mg, 0.07 mmol, 1 equiv), dimethylformamide (0.84 mL), and naphthalene (25 mg, as internal standard). The mixture was homogenized and degassed by sparging with nitrogen for 30 min. The nitrogen purge was stopped, and the flask was sealed with a nitrogen-filled balloon. An aliquot for GC-FID analysis was taken, and the flask was immersed in a 70 °C oil bath. Over the course of polymerization, several aliquots were taken for GC-FID analysis to determine monomer conversion. The reactivity ratios were extracted from the resulting Jaacks plot.

### Synthesis of Poly(oligo(ethylene glycol) methyl ether acrylate) (POEGA) Macro-RAFT Agent

A 250 mL three-neck flask equipped with a magnetic stirrer was charged with AIBN (91 mg, 0.56 mmol, 0.07 equiv), CDT (2646 mg, 8.33 mmol, 1 equiv), OEGA (average *M*_n_ = 480 g/mol) (40.00 g, 83.33 mmol, 10 equiv), toluene (184 mL), and trioxane (400 mg, as internal standard). The mixture was homogenized by stirring and degassed by sparging with nitrogen for 30 min. After degassing, an aliquot for NMR analysis was taken and the flask was placed in a 70 °C oil bath. During the reaction, several aliquots for NMR analysis were taken to follow the extent of the reaction. The integrals of the acrylate resonances were compared to the trioxane resonance. After 360 min, the flask was taken out of the oil bath and cooled to room temperature. The polymer was precipitated three times in hexane and dried overnight in a vacuum oven at 40 °C. A yellow, transparent, and viscous liquid was obtained. The results of the POEGA macro-RAFT agent are summarized in Table S1. Since the molecular weight found in ^1^H NMR corresponds well with the theoretical molecular weight, a high end-group fidelity is obtained. The overlay of the assigned ^1^H NMR spectra of the monomer and purified polymer in CDCl_3_ is shown in Figure S1.

### Small-Scale Screening Emulsion Polymerization

All of the small-scale emulsion polymerization reactions were performed in a similar fashion. The results are presented in the SI, section 2. In an exemplary synthesis (Table S5, Latex2OA), a 25 mL Schlenk flask equipped with a magnetic stir bar was charged with 4CPA (300 mg, 1.973 mmol, 50 equiv), 2OA (945 mg, 5.130 mmol, 130 equiv), IBOA (164 mg, 0.789 mmol, 20 equiv), the POEGA macro-RAFT agent (164 mg, 0.04 mmol, 1 equiv), VA-044 (12.8 mg, 0.039 mmol, 1 equiv), distilled water (2.114 mL), and naphthalene (60 mg) as the internal standard. While stirring at 600 rpm, the mixture was sparged with nitrogen for 30 min, and then the flask was sealed with a nitrogen-filled balloon. A sample was taken for GC-FID measurement. The flask was placed in a 50 °C oil bath. Regular samples were taken for GC-FID to determine the monomer conversion. At the moment of rapid increase in the monomer conversion, in this example, after about 85 min, the mixture changed from an off-white turbid to a milky white emulsion with a blue haze, indicating sub-100 nm particles. When the monomer conversion plateaued, in this example, after 190 min, the flask was cooled to room temperature, and the latex was stored at 4 °C.

### Scaled Up Emulsion Polymerization

All of the scaled up emulsion polymerization reactions were performed in a similar fashion. In this example, the synthesis of Latex25 (Table 1) is described. The emulsion polymerization was performed in a 500 mL double-walled cylindrical glass reactor equipped with a mechanical paddle stirrer with three holes. To the reactor were added 4CPA (16.63 g, 109.40 mmol, 50 equiv), THFA (34.19 g, 218.90 mmol, 100 equiv), IBOA (22.79 g, 109.40 mmol, 50 equiv), the POEGA macro-RAFT agent (10.15 g, 2.189 mmol, 1 equiv), and distilled water (110 mL). The mixture was degassed by sparging with nitrogen for 30 min. Then, the sparging was stopped, and a low nitrogen flow was applied on the overhead space of the reactor. While stirring at 250 rpm, oil regulated by a thermostat at 53 °C was pumped through the double wall of the reactor to bring the inside temperature to 50 °C. After the temperature was stable, VA-044 (708 mg, 2.189 mmol, 1 equiv) was added at once to the reaction mixture, marking the start of the polymerization. After 220 min, the reaction was stopped by cooling the reactor to room temperature. The latex was filtered over a 190 μm nylon filter, and an off-white, turbid, and viscous latex was obtained. The latex was stored at 4 °C. Yield: 173.67 g (89.1%).

**Table 1:** Properties of the Scaled Up Latexes with 25 mol % 4CPA in the Feed

code	THFA feed (mol %)	IBOA feed (mol %)	2OA feed (mol %)	*T*_g_ (°C)	MFFT (°C)	gel content (wt %)	size DLS (nm)	PDI	size Cryo-TEM (nm)	ζ (mV)	solids (%)
Latex10	65	10	0	8	0	93	549	0.264	350 ± 111	–13.2	34.6
Latex25	50	25	0	19	13.7	90	187	0.010	177 ± 28	–14.6	47.1
Latex50	50	50	0	56	46	92	115	0.090	94 ± 10	–7.5	45.1
Latex2OA	0	10	65	–21	0	95	136	0.142	82 ± 19	–7.4	42.6

MFFT = Minimum Film Formation Temperature.

For the synthesis of the other scaled up latex syntheses, the following reagent quantities were used.

#### Latex10

4CPA (16.39 g, 107.82 mmol, 50 equiv), THFA (43.78 g, 280.32 mmol, 130 equiv), IBOA (8.98 g, 43.13 mmol, 20 equiv), the POEGA macro-RAFT agent (10.00 g, 2.16 mmol, 1 equiv), distilled water (161 mL), and VA-044 (697 mg, 2.16 mmol, 1 equiv). Yield: 229.12 g (95.0%).

#### Latex50

4CPA (16.39 g, 107.81 mmol, 50 equiv), THFA (16.84 g, 107.81 mmol, 50 equiv), IBOA (44.92 g, 215.63 mmol, 100 equiv), the POEGA macro-RAFT agent (10.00 g, 2.16 mmol, 1 equiv), distilled water (117 mL), and VA-044 (697 mg, 2.16 mmol, 1 equiv). Yield: 179.49 g (87.1%).

#### Latex2OA

4CPA (16.63 g, 109.45 mmol, 50 equiv), 2OA (52.44 g, 284.57 mmol, 130 equiv), IBOA (9.12 g, 43.78 mmol, 20 equiv), the POEGA macro-RAFT agent (10.15 g, 2.19 mmol, 1 equiv), distilled water (117 mL), and VA-044 (708 mg, 2.19 mmol, 1 equiv). Yield: 144.48 g (70.3%). Some coagulant was retrieved after filtration (15.65 g, 17.6 wt % based on solids), and material loss on the reactor walls explains the low yield.

### Freestanding Film Preparation

Three milliliters of latex was diluted to 12 mL with distilled water and cast in a circular PTFE evaporation dish with a diameter of 75 mm. The films were dried for 2 days in air. The dish was then transferred to a nitrogen-filled Dymax ECE 2000 UV chamber equipped with a 400 W metal halide UVA lamp. After 20 min of irradiation, the film was turned upside down and irradiated for another 20 min to achieve a homogeneous curing. The distance from the sample to the lamp was 18 cm. The final dry film thickness was about 0.2 mm.

The CNC-reinforced composite films were prepared as follows: First, a 3 wt % CNC dispersion was prepared by manually stirring the CNC powder in water until a homogeneous viscous mixture was obtained. This step was followed by 30 min of sonication in a Branson 3800 sonication bath at room temperature, resulting in a mixture with considerably lower viscosity. An accurate amount of CNC dispersion was thoroughly mixed with 3 mL of diluted latex to a total amount of 12 mL. The mixture was cast in a PTFE evaporation dish with a diameter of 75 mm. The mixing ratio of CNC dispersion and latex was determined based on a targeted solid ratio in the final dried film. After drying at room temperature for 3 days, the films were irradiated in the UV chamber as described above. The final dry film thickness was about 0.2 mm.

### Film Preparation

The latexes were directly applied on Leneta cards and cold-rolled R-46 steel Q-panels (obtained from Benelux Scientific BV) using a manual bar coater with a 120 μm gap. Films on bleached printer paper with an average weight of 78 ± 1 g/m^2^ were applied using the manual bar coater at specified wet layer thicknesses. The films were dried at room temperature for 1 day. Films from Latex50 were dried in an 80 °C oven for 30 min because of the high minimum film formation temperature. After drying, the films were transferred to a Dymax ECE 2000 UV chamber under nitrogen flow and irradiated using a 400 W metal halide UVA lamp. The distance between the sample and the lamp was 18 cm. The films were irradiated for a specified amount of time. The reference film based on CHP204 was only dried but was not subjected to UV irradiation since this is not a curable latex.

Details on the analytical methods and characterization techniques of the films can be found in Supporting Information (SI), section 1.

## Results and Discussion

### Synthesis and Characterization of 4CPA Latexes

**Figure 2:**
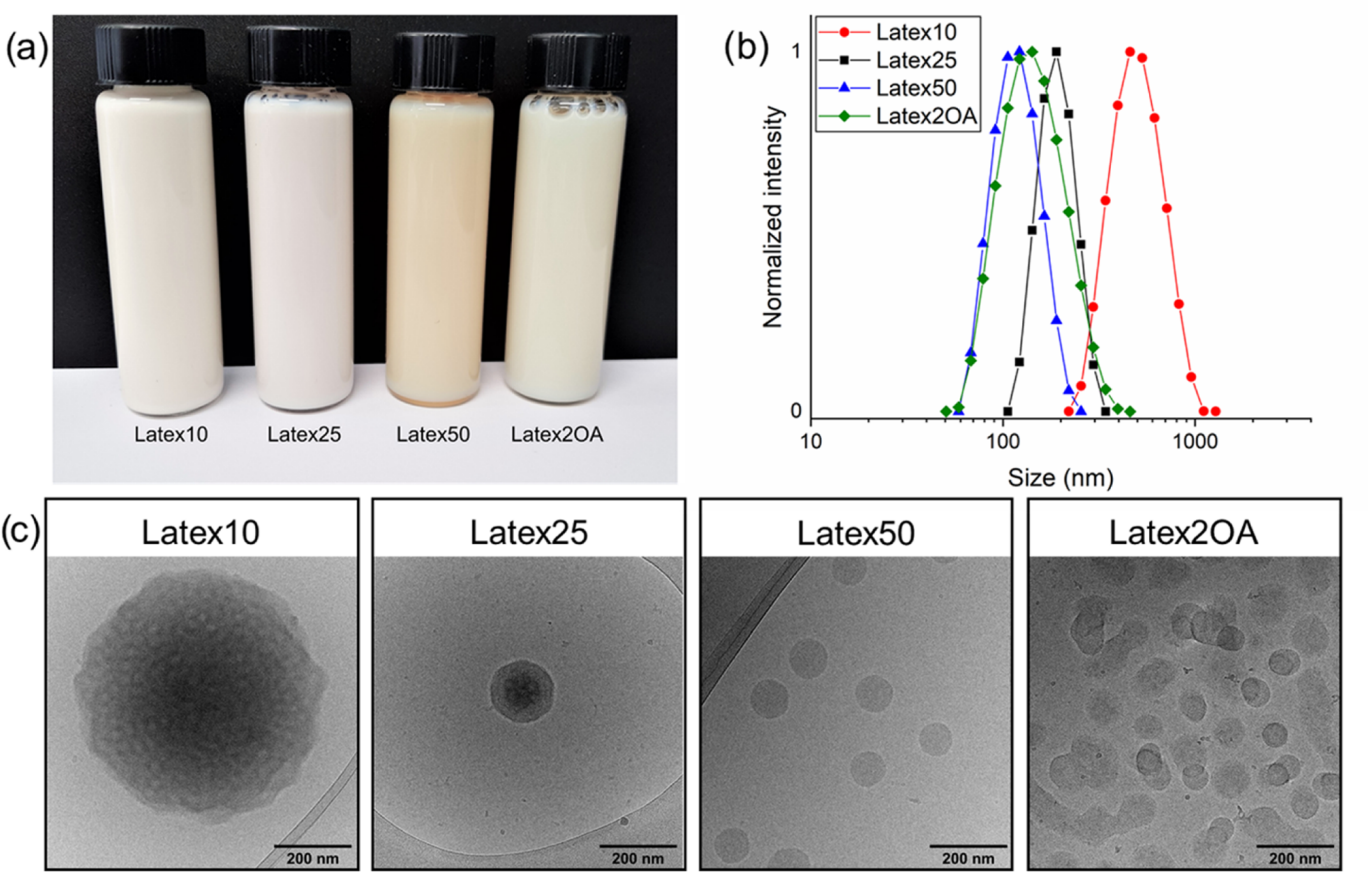
(a) Photograph depicting the appearance of the synthesized latexes, (b) Overlay of the DLS particle size distributions, and (c) Representative cryo-TEM images of the synthesized latexes as presented in Table 1.

First, the desired reaction conditions for emulsion polymerization of 4CPA latexes on a small scale, reaction kinetics, and investigation toward the copolymerization of 4CPA with the renewable comonomers THFA, IBOA, and 2OA were established. Polymer latexes containing intact cyclopentenone side groups were obtained according to ^1^H NMR spectroscopy. The details can be found in the SI, section 2. In short, an optimum of 25 mol % 4CPA in the monomer feed was found, yielding stable, high solid latexes. Subsequently, we proceeded with upscaling the most promising polymerizations, latexes 10, 25, 50, and 2OA (Table 1). The reactions were performed on a scale of about 200 mL to produce sufficient material for the evaluation of coated substrates and freestanding film properties. Latex10, 25, and 50 were made in order to evaluate the effect on the amount of IBOA in the monomer feed and, thus, the effect of polymer *T*_g_ on the film properties. To further reduce the *T*_g_ to below room temperature, THFA was replaced by 2OA (latex2OA), a proposed renewable alternative to the fossil-based 2-ethylhexyl acrylate. The appearance of the latexes was opaque, and the color ranged from off-white to pale yellow (Figure 2a). In general, latexes having a solid content between 34.6 and 47.1 wt % were obtained, which is in the range of commercial latex binder products.^25^

From the results summarized in Table 1, it is clear that the amount of IBOA and 2OA in the monomer feed drastically influences the *T*_g_ of the latex polymer. The *T*_g_ increases from 8 to 56 °C with an IBOA feed of 10 and 50 mol %, respectively. Since the minimum film formation temperature (MFFT) is mainly dictated by the *T*_g_, this also increases with an increasing amount of IBOA in the monomer feed. Samples Latex10, Latex25, and Latex2OA have an MFFT below 15 °C and are expected to form a homogeneous layer at room temperature. Latex50, however, has an MFFT of 46 °C and, thus, elevated temperatures are required during drying to achieve coalescence of the latex particles.

As was observed in the small-scale screening reactions, the IBOA feed fraction also clearly affected the particle size distribution. According to dynamic light scattering (DLS), a relatively large average particle size of 549 nm and a broad dispersity were obtained at a low IBOA feed of 10 mol % in Latex10. At higher feeds of 25 and 50 mol % IBOA, the particle size dropped drastically to 187 and 115 nm, respectively. A similar small particle was observed for Latex2OA. It is possible that the relatively hydrophobic IBOA and 2OA influence the self-assembly during the PISA process in a different way compared to the more hydrophilic THFA.^26^ The cryo-TEM images in Figure 2c clearly show the difference in the morphology of the different latex particles. Large and coarse particles were obtained in the Latex10 sample, possibly consisting of coagulation of multiple smaller particles. Core–shell type particles are observed in Latex25, whereas in Latex50, homogeneous spherical particles were obtained. In Latex2OA, small, spherical, and partially coagulated particles were obtained, which are also represented in the overview of the DLS distributions (Figure 2b), where Latex2OA shows a rather broad distribution. In general, the sizes observed in cryo-TEM correspond well with those measured by DLS (Table 1). Various morphologies, including core–shell structures, have been reported for the RAFT PISA process, and the morphology has been shown to depend on a variety of conditions, such as the monomer and polymer structures.^27,28^ In the case of core–shell type particles, typically hydrophobic segments orient themselves in the core, while hydrophilic segments are present in the shell.^29^

The main mode of particle stabilization is steric repulsions of the nonionic POEGA macro-RAFT shell. This is evident from the measured ζ-potential values, which are close to zero (Table 1). In contrast to linear macro-RAFT agent stabilizers, the brush-type POEGA is known to exhibit strong steric repulsion due to the densely populated side chains that are covalently bonded to the particles.^30^ Indeed, the emulsions were stable for several months of storage. No visual sedimentation or coagulation was observed, and the particle size remained the same after 11 months of storage at 4 °C according to DLS (Table S7). The latexes were subjected to several stabilization tests to evaluate the resistance against freeze–thaw, the addition of one equivalent of salt solution (1 M NaCl and 0.1 M MgSO_4_), and solvent (ethanol). The latexes were evaluated visually for any macroscopic phase separation. None of the latexes showed any phase separation after the addition of salt solution or solvent (Table S8). High resistance toward the addition of ions is characteristic of sterically stabilized latexes.^31^ Latexes 10, 25, and 50 were macroscopically stable after the freeze–thaw step, whereas Latex2OA coagulated and showed phase separation. DLS measurements before and after freeze–thawing showed that only Latex50 was completely resistant, while the other latexes showed an increased particle size and broader distribution (Table S7). A higher increase in the particle size was observed for the latexes with a low *T*_g_, suggesting that coagulation of the soft particle cores is the cause of the increase in particle size during the freeze–thaw step.

The amount of unreacted macro-RAFT agent remaining in the latexes was determined gravimetrically from the solids that remained from the supernatant after centrifugation for 2.5 h at 15 000 rpm (Table S9). ^1^H NMR spectroscopy suggests that the majority of said residue consists of the POEGA macro-RAFT agent, together with a small amount of unidentified impurities. The amount of free surfactant in the synthesized latexes was calculated to be between 35 and 46%. The formation of a small amount of dead chains is expected during RAFT polymerization of OEGA, which partially contributes to the amount of free surfactant. Incomplete chain-transfer efficiency would explain the remaining free surfactant. The free polymeric surfactant can also be beneficial during the formulation and application of latex since it lowers the surface tension and aids with leveling and wetting on the desired surface.^32^ The surface tension of the latexes ranged between 44.8 and 49.3 mN/m, which is significantly lowered compared to that of pure water at 72.5 mN/m (Table S9 and Figure S9).

Rheological measurements confirmed the low viscosity of the latexes of below 100 Pa•s, with shear thinning behavior (Figure S10). The viscosity of latex is governed by many factors, including particle size, particle morphology, and solid content.^33^ Since multiple factors differ between the latexes in this work, no conclusions can be drawn regarding the influence on the rheological properties.

### Film Formation, UV Curing, and Tensile Properties of the Freestanding Films

**Figure 3:**
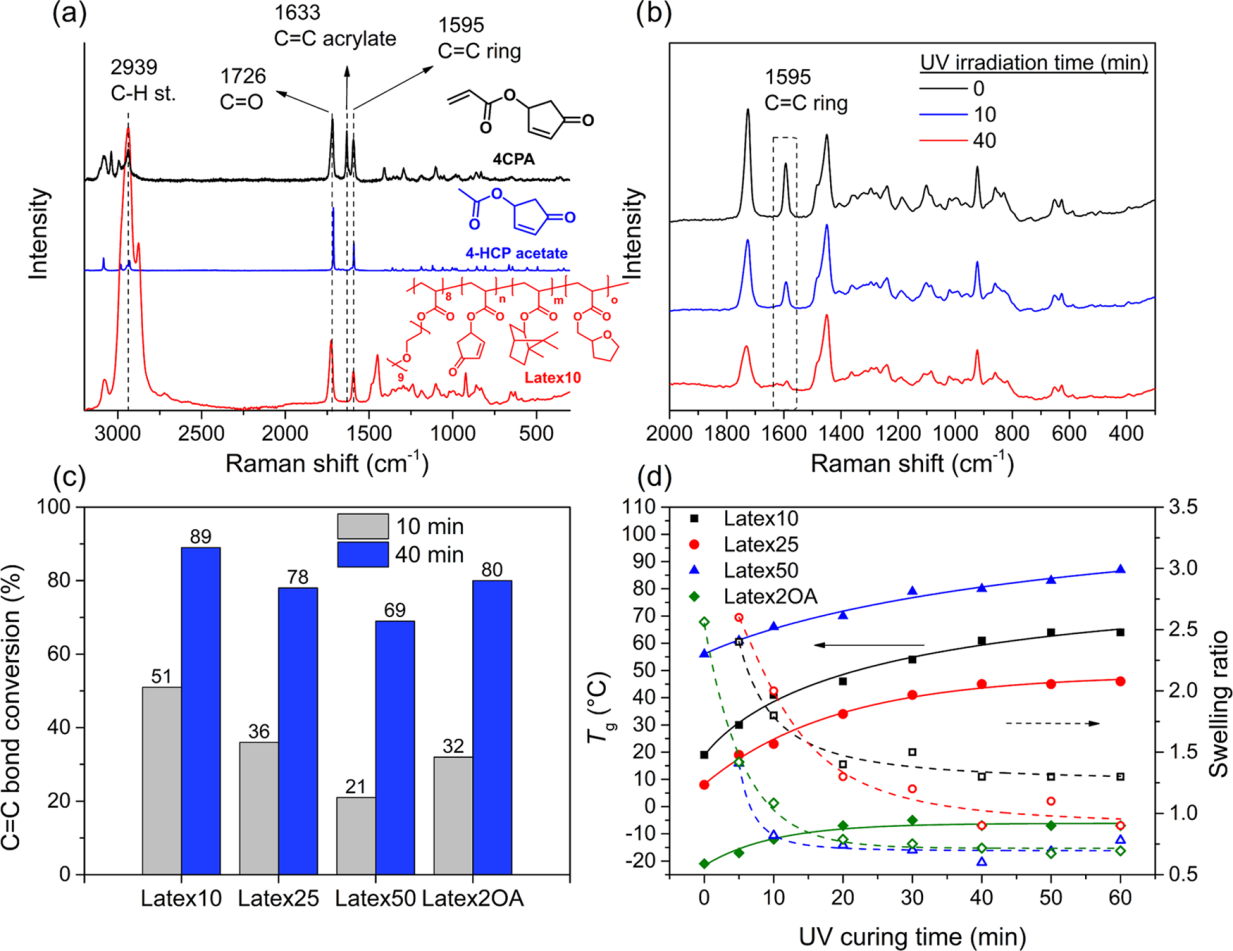
Characterization of the extent of cross-linking of the latex freestanding films. (a) Overlay of the Raman spectra of 4CPA, 4HCP acetate, and Latex10 between 300 and 3300 cm^–1^. (b) Overlay of the Raman spectra of Latex10 between 300 and 2000 cm^–1^ after 0, 10, and 40 min in the UV chamber. The spectra were normalized against the band at 2939 cm^–1^ (C–H stretching). (c) C=C double bond conversion from the cyclopentenone group for each latex. (d) The influence of UV curing time on the *T*_g_ (closed symbols) and swelling ratio (open symbols) in THF for each latex.

Freestanding films of the latexes presented in Table 1 were prepared by casting the latex in PTFE dishes and drying, followed by irradiation in a UV chamber for 40 min. This yielded optically clear and homogeneous cross-linked films. Since cross-linking occurs by photocyclodimerization of the pendent cyclopentenone double bonds, no addition of cross-linker or photoinitiator was required.^17^ The extent of cross-linking was followed by the disappearance of the cyclopentenone double bond by using Raman spectroscopy. First, the unmodified film of Latex10 was analyzed. In Figure 3a, the overlay of the Raman spectra belonging to 4CPA, 4HCP acetate, and Latex10 are shown. By comparing the spectra, it can be determined that the peaks at 1726, 1633, and 1595 cm^–1^ belong to the carbonyl, acrylate, and cyclopentenone double bond, respectively. As a result of UV irradiation, the C=C double bond corresponding to the cyclopentenone group disappears relative to the signal at 2939 cm^–1^ (C–H stretching). By measuring the integral of the C=C double bond signal and assuming that at 0 min UV irradiation, 100% of the bonds are intact, the conversion can be calculated (Figure 3c). After 10 min of UV irradiation, conversions between 21 and 51% are obtained. After 40 min, the conversion is increased to between 69 and 89%. During the UV curing, the temperature of the films reached up to 90 °C, which is above the *T*_g_ of all of the cross-linked freestanding films (Figure 3d).

The *T*_g_ was visible in the DSC traces but appeared as broad transitions. A general trend of gradual *T*_g_ increase during UV curing was observed and reached a plateau after about 40 min. The effect of the UV irradiation time on the swelling ratio also indicated cross-linking. The swelling ratio steadily decreases as a result of the UV irradiation time, also plateauing after about 40 min for every latex film.

The latexes showed a considerably high gel content prior to film formation and cross-linking. It was reported previously that in the solvent copolymerization of 4CPA, the dispersity increased exponentially when approaching 100% monomer conversion.^17^ Therefore, at a high monomer conversion of the latexes reported herein, gel formation is expected. Despite the relatively high gel content of the latexes (Table 1), homogeneous films with good mechanical performance were obtained after UV curing. This suggests that sufficient coagulation and migration of non-cross-linked or dangling chains between the particles took place to achieve interparticle cross-links and a macroscopically robust film.

**Figure 4:**
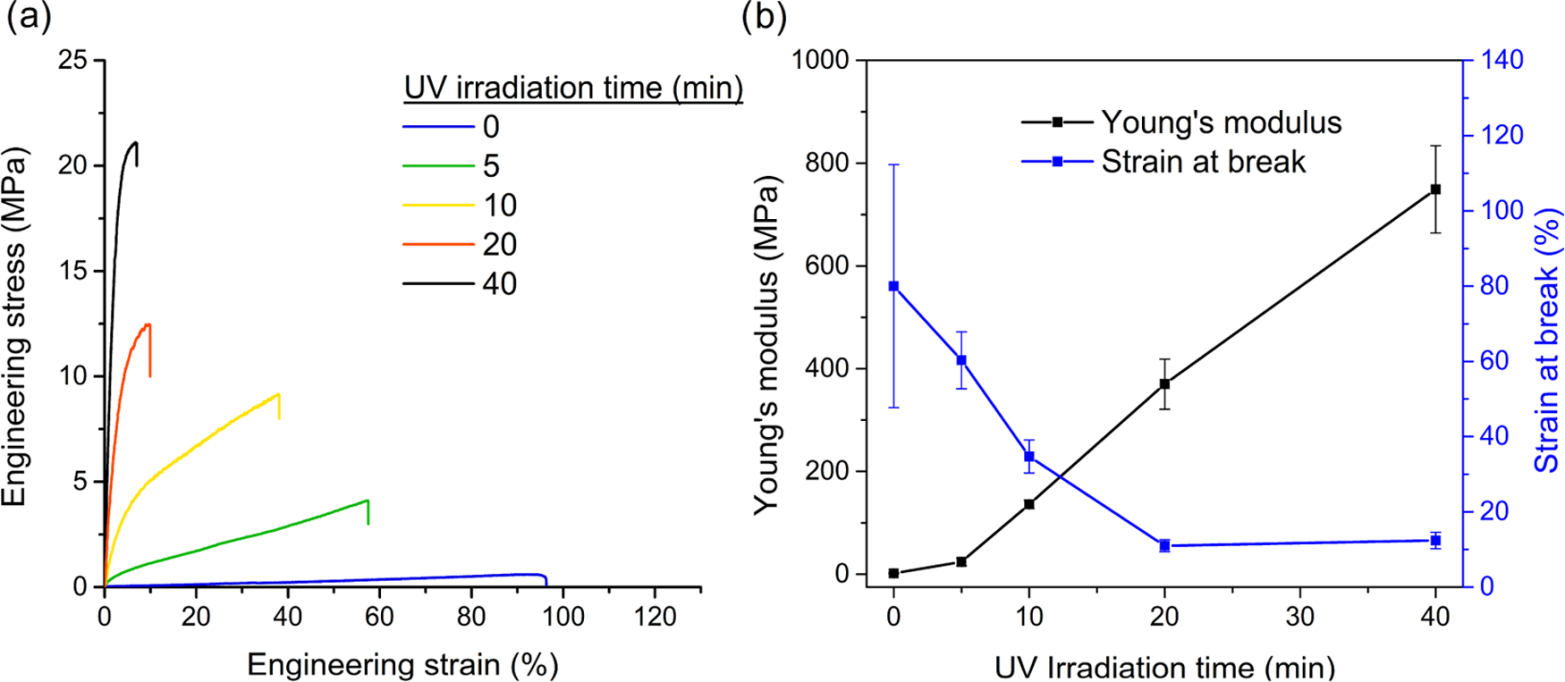
(a) Representative tensile curves of the freestanding films from Latex10 cured at different times in the UV chamber. (b) Effect of the UV curing time on the Young’s modulus and Strain at break of freestanding films from Latex10.

The results of the tensile tests of the freestanding films are reported in Table S10. The high gel content (98.2–99.8 wt %) of the cross-linked films from latexes 10, 25, 50, and 2OA corroborates witha high extent of cross-linking and that potential residual monomers will have reacted to form insoluble products during the UV irradiation step. The monomer composition had a significant influence on Young’s modulus and ultimate tensile strength. Latex10, 25, and 50 had Young’s moduli of 749 ± 85, 1052 ± 47, and 1248 ± 117 MPa, respectively. The Young’s modulus was drastically decreased to 63 ± 16 MPa using the soft monomer 2OA to obtain films with higher flexibility. The strain at break for all freestanding films was relatively low, between 5.4 and 13.0%, which can be expected for extensively cross-linked polymers. Indeed, films with a lower degree of cross-linking yielded a lower Young’s modulus but a much higher strain at break. The extent of cross-linking was controlled by limiting the UV irradiation time. In Figure 4a, the stress–strain curves of freestanding films from Latex10 with different UV irradiation times are presented. The numerical data is summarized in Table S11. The unmodified films were ductile and fragile, with a strain at a break of 80 ± 32.3%. Increasing the UV irradiation time results in an increase in Young’s modulus and ultimate tensile strength while the strain at the break decreases (Figure 4b). In this way, the properties of the freestanding films can be facilely tuned by controlling the UV irradiation time. For example, high-stiffness films might be needed in applications where scratch resistance is required, whereas higher flexibility is generally desired for paper or wood coating applications.^34^

Additional evidence of the relationship between tensile properties and UV irradiation time was provided by comparison to a reference latex containing 10 mol % 4CPA instead of 25 mol %. The details of this latex are summarized in Table S12. Freestanding films that were irradiated for 40 min had tensile properties similar to the films from Latex10 that were irradiated for 5 min (Figures 4 and S11). In order to test if prolonged UV irradiation times would result in improved tensile properties, one freestanding film prepared from Latex25 was irradiated for a total of 60 min (30 min on each side) (Table S11). This resulted in similar tensile properties as 40 min UV irradiation time (Table S10). Similarly, a film with three times the thickness (0.61 mm) was prepared and irradiated for a total of 40 min. The Young’s modulus and ultimate tensile strength were significantly reduced, while the strain at break was increased (Table S11). This indicates incomplete conversion of the 4CPA bond due to the limited penetration depth of the UV light, resulting in a less dense network. Typically, thin films are more rapidly and homogeneously cured by UV light compared to thick films. In the next section, the results of testing on coated substrates are discussed. The coatings are significantly thinner than the freestanding films. As a consequence, it can be assumed that they are cross-linked to completion after the same UV irradiation time.

The thermal stability of the unmodified and cross-linked films was investigated by TGA. The temperature at 5 wt % weight loss was between 225 and 259 °C, and the residue at 700 °C was between 5.3 and 8.7% for all films (Table S13). No significant influence of UV curing on the thermal stability was observed (Figure S12).

### Coated Substrates

To investigate the application of polymer latexes as potential binders in coating formulations, latex films were applied on steel substrates and Leneta cards to evaluate the unformulated film properties. The latexes were applied with a wet film thickness of 120 μm, dried, and cured in a UV chamber. The contact angle, gloss, solvent and water resistance, hardness, blocking resistance, and adhesion were evaluated on the cured films with a dry thickness of between 24 and 46 μm (Table 2). For comparison, commercial styrene-acrylate latex CHP204 was used as a reference. CHP204 is typically used as a binder in paper coating formulations.

**Table 2:** Evaluation of the Film Properties of the 4CPA Latexes and Commercial Reference Material CHP204

code	curing time (min)	dry film thickness (μm)	contact angle H_2_O (deg)	gloss 60° (GU)	MEK double rub	H_2_O double rub	pencil^a^ hardness	blocking resistance^b^	cross-cut adhesion
Latex10	0	30 ± 5	11.6 ± 1.0	34.7 ± 0.1	5 ± 2	>200	3B	C1	5B
	5	24 ± 5	54.8 ± 0.7	N/A	38 ± 11	>200	H	B0	3B
	10	26 ± 5	59.5 ± 0.7	N/A	140 ± 59	>200	H	B0	3B
	40	30 ± 1	64.3 ± 0.5	31.4 ± 0.9	>200	>200	2H	A0	5B
Latex25	0	35 ± 2	17.3 ± 1.7	49.9 ± 0.3	5 ± 0	>200	3B	A0	5B
	40	46 ± 2	67.6 ± 0.5	35.5 ± 0.4	>200	>200	4H	A0	5B
Latex50	0	34 ± 3	36.3 ± 1.4	79.5 ± 0.8	6 ± 0	>200	HB	A0	4B
	40	40 ± 2	76.8 ± 0.5	92.0 ± 1.6	>200	>200	5H	A0	5B
Latex2OA	0	31 ± 2	60.0 ± 0.7	75.8 ± 0.9	12 ± 5	93 ± 8	<6B	D5	5B
	40	28 ± 6	96.3 ± 0.3	84.9 ± 0.5	>200	>200	H	C0	5B
CHP204	0	16 ± 2	91.1 ± 0.1	78.9 ± 1.2	7 ± 1	>200	HB	D4	5B

All characterizations were performed on the coatings using steel substrates, except the blocking resistance, which was performed on Leneta cards.

a Ranging between 6B (soft) to 6H (hard).

b Ranging between A0 (excellent blocking) to F5 (poorest blocking properties).

The film properties were evaluated before and after UV irradiation. The results in Table 2 show that UV irradiation improves all of the evaluated properties. First, the solvent resistance represented by the amount of double rubs with methyl ethyl ketone (MEK) is drastically increased. All unmodified films showed surface damage after several double rubs with MEK, while all fully cross-linked films remained intact after at least 200 MEK double rubs. Coatings that show no defects after 200 MEK double rubs are considered solvent-resistant. Second, the pencil hardness improved after UV irradiation. The cured films had a pencil hardness between 2H and 5H, with a pronounced effect of the *T*_g_ of the initial latex polymer. A higher *T*_g_ resulted in a higher pencil hardness. In the development of water-borne coatings, it remains a challenge to achieve both good blocking resistance and film formation at ambient temperature. The low *T*_g_ of the binder required for particle coalescence also impairs the blocking resistance of the resulting film.^35^ After extensive cross-linking, however, the *T*_g_ of the polymer is increased and chain diffusion is restricted by the covalent cross-links. Therefore, the cross-linked films studied in this research had good blocking resistance properties, as indicated in Table 2. The blocking resistance was poor for the unmodified films from Latex10 and 2OA, but complete separation of the films without surface damage was achieved after the UV irradiation step. The UV irradiation also improved the gloss of most films, resulting in medium to high gloss values. Overall, the films showed good adhesion to steel substrates.

**Figure 5:**
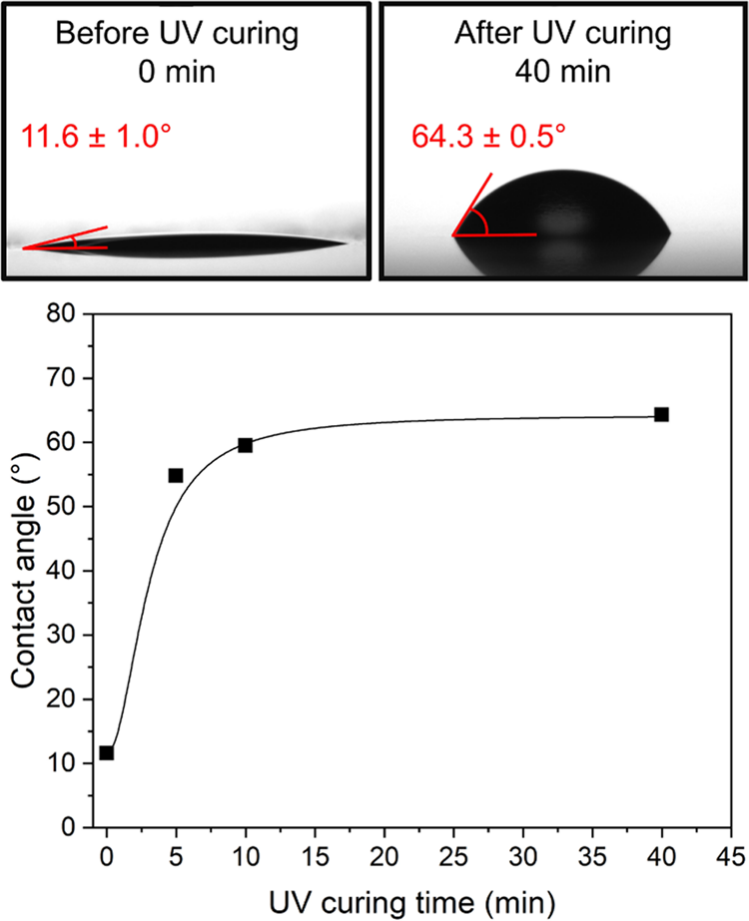
Effect of the UV curing time of a film of Latex10 on the surface contact angle with water. The standard deviation of each measured point was below 1.

Finally, the contact angle of water on the films was determined. As expected, increasing the amount of the hydrophilic THFA monomer and decreasing the amount of hydrophobic monomers IBOA and 2OA results in a decrease in the contact angle. Furthermore, the films after UV irradiation had a significantly higher contact angle than before (Figure 5). In the case of films from Latex10, the contact angle increased from 11.6 ± 1.0° before UV curing to 64.3 ± 0.5° after UV curing. This drastic change indicates that UV curing plays an important role in the film formation step. The change in the contact angle as a result of the UV curing could be explained by topological or chemical changes on the surface as a result of the photocyclodimerization reaction. For example, the rearrangement of the hydrophilic and hydrophobic segments. Alternatively, the UV curing process could mitigate surface defects that could otherwise affect the contact angle with water.

The cross-linked films of the latexes reported herein show comparable properties to those of the reference latex CHP204, except for the MEK double rub and blocking resistance. The MEK double rub and blocking resistance are indicative of cross-linking, and therefore CHP204 performs worse in these tests since the reference film is based on a non-cross-linkable polymer.

### Barrier Layers for Paper Applications

Layers of latex2OA were applied on paper substrates and UV cured to investigate the potential application as oil and water barrier layers for paper applications. Since the UV curing of the applied films leads to a cross-linked network with high solvent resistance (>200 MEK double rubs) and high contact angle with water (96.3°) (Table 2), the ability to block oil and moisture is promising. Furthermore, this latex exhibits a low *T*_g_, facilitating film formation at room temperature and maintaining flexibility on flexible substrates after UV curing. Latex2OA was applied on bleached uncoated paper with various wet layer thicknesses, resulting in a weight of between 3.3 and 27.6 g/m^2^ of the dried and UV-cured film. The results are summarized in Table 3. Oil barrier properties were assessed by using the KIT oil and grease resistance test. The KIT test determines whether a coating on paper can act as a barrier to a solvent mixture consisting of heptane, toluene, and castor oil for 15 s. A series of 12 solvent mixtures are evaluated, with number 1 being pure castor oil and number 12 a mixture of toluene:heptane (45:55) being the most harsh mixture of the test. All of the applied layers showed the highest KIT test number of 12, except for the two lowest-weight layers corresponding to the single layers with a dry coating weight of 3.3 and 6.2 g/m^2^. Nonetheless, these films still resulted in high KIT test numbers of 8.5 and 9.5, respectively. The reference latex CHP204 scores slightly higher in the KIT test in comparison to Latex2OA.

**Table 3:** Results of the Paper Substrates Coated with Latex2OA with Various Wet Layer Thicknesses and Single and Double Layer

			Latex2OA		CHP204	
entry	wet layer thickness (μm)	single or double layer	dry coating weight	KIT test no. (g/m^2^)	dry coating weight (g/m^2^)	KIT test no.
1	30	single	3.3 ± 1.0	8.5 ± 0.5	2.5 ± 0.8	9.5 ± 0.5
2	30	double	6.8 ± 0.7	12 ± 0	6.9 ± 1.0	12 ± 0
4	90	single	6.2 ± 1.7	9.5 ± 0.5	8.3 ± 1.3	12 ± 0
5	90	double	18.6 ± 2.7	12 ± 0	14.5 ± 2.1	12 ± 0
7	120	single	10.3 ± 2.0	12 ± 0	10.3 ± 2.5	12 ± 0
8	120	double	27.6 ± 5.6	12 ± 0	20.1 ± 5.1	12 ± 0

**Figure 6:**
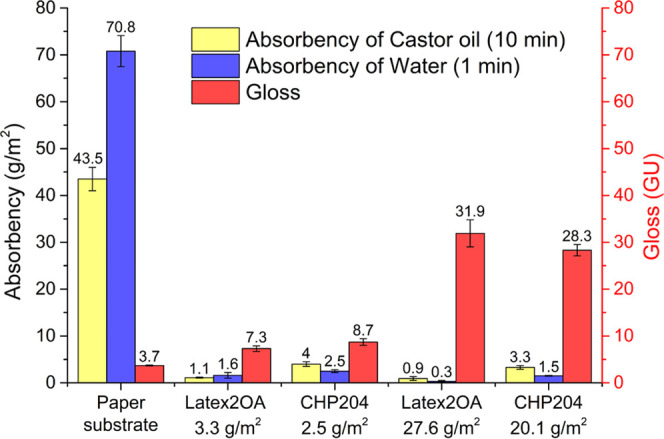
Cobb-Unger test results after exposure to castor oil (10 min) and water (60 s) for the paper substrate and the substrates coated with Latex2OA and CHP204. Gloss values of the paper substrate and the coated substrates. Dry coating weights are given.

Further oil and water barrier properties were characterized by using the Cobb-Unger test method (Figure 6). The paper substrate was compared to the two films with the lowest and highest dry coating weight of Latex2OA. In both cases, a drastic decrease in the oil and water absorbency was observed for the coated substrates. The values obtained for the coating with a weight of 3.3 are 1.1 g/m^2^ for castor oil after 10 min exposure and 1.6 g/m^2^ for water after 1 min exposure. There was only a slight improvement in the oil and water absorbency of the coating, with a weight of 27.6 g/m^2^, resulting in absorbency values of 0.9 and 0.3 g/m^2^ for castor oil and water, respectively. This small difference indicates that an application of 3.3 g/m^2^ already results in drastically improved properties under the evaluated conditions. From a commercial point of view, low coating weights are desired, while a Cobb value of close to zero is favorable.^36^ In Table S14, the Cobb60 water absorption values of various paper coatings in the literature are summarized. The values for the material reported herein are lower compared to all references while also exhibiting a low coating weight. In comparison to the reference latex CHP204, the water and castor oil absorbencies are lower at a comparable coating weight (Figure 6).

The gloss of the final surfaces was also improved after coating with Latex2OA. The single wet layer of 30 μm gave only a slight increase in the gloss at 60° from 3.7 GU for the paper substrate to 7.3 GU for the coated substrate. The most glossy films were obtained with the thickest wet layer of 120 μm (double layer), resulting in a gloss at 60° of 31.9 GU (Figure 6).

### Properties of Cellulose Nanocrystal (CNC) Reinforced Latexes and Freestanding Films

The covalently bonded PEG hydrophilic stabilizer is potentially compatible with fillers that can form hydrogen bonds to produce composite materials. Cellulose nanocrystals (CNC) are of high interest due to their biocompatibility, low toxicity, applicability in food applications,^37^ and high aspect ratio in order to mechanically reinforce polymer materials.^38^ PEG is able to form hydrogen bonds with the free hydroxyl groups that are present on the surface of CNC’s.^39^ Therefore, the same effect is expected for the POEGA macro-RAFT agent. Latex polymers functionalized with carboxylic acid groups have been shown to be successfully reinforced with CNC due to the interactions between the carboxylic acid and hydroxyl groups.^40^ In here, the latexes were simply mixed with a 3 wt % CNC dispersion in water in various ratios. After casting, drying, and UV curing, CNC-reinforced freestanding films were obtained with various ratios of polymer and CNC.

**Figure 7:**
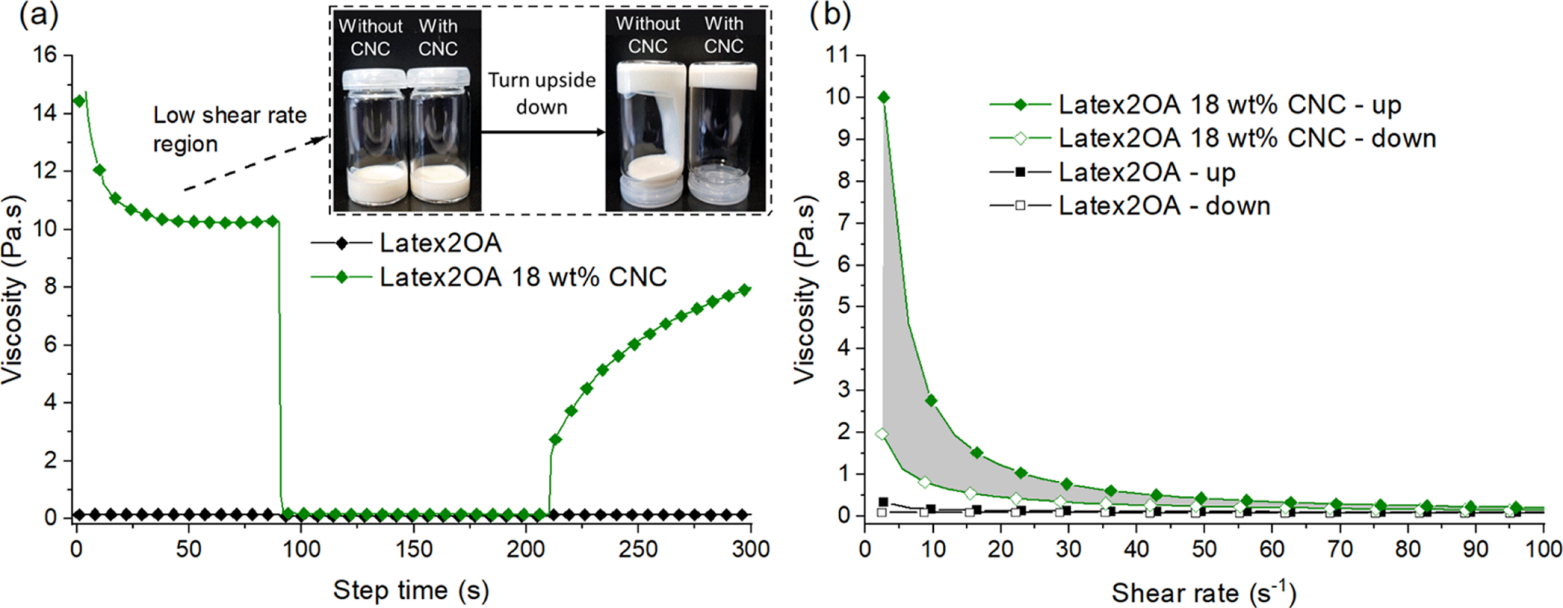
(a) 3ITT curves of Latex2OA and the 18 wt % CNC mixture alternating between a shear rate of 1 s^–1^ for 90 and 100 s^–1^ for 120 s. (b) Hysteresis loop curves of Latex2OA and the 18 wt % CNC mixture with a step time of 60 s.

The first indication of the presence of an interaction between the CNC and latex was observed during the mixing of the latex and CNC dispersion. A mixture was obtained with drastically increased viscosity relative to those of the original components. This indicates a bridging interaction between the CNC’s and the PEG hydrophilic stabilizer shells through hydrogen bonding. Using the rheological Three Interval Thixotropy Test (3ITT) and hysteresis loop measurements, it was confirmed that the latex CNC mixtures had strong shear thinning properties (Figures 7 and S5). Further details can be found in section 3 of the SI. The rheological interactions observed here are similar to the interaction between some surfactants and associative thickeners, as described previously.^41^

The latexes mixed with CNC dispersion were cast and dried at room temperature to obtain freestanding films. After 40 min of UV irradiation, clear and homogeneous films were obtained. From the freestanding films, dog bone-shaped test specimens were stamped, and the tensile properties were measured. In addition, to develop an understanding of the interaction with water as a result of the hydrophilic CNC filler, water uptake, contact angle, and gel content measurements were performed. The numerical data is summarized in Table S10.

**Figure 8:**
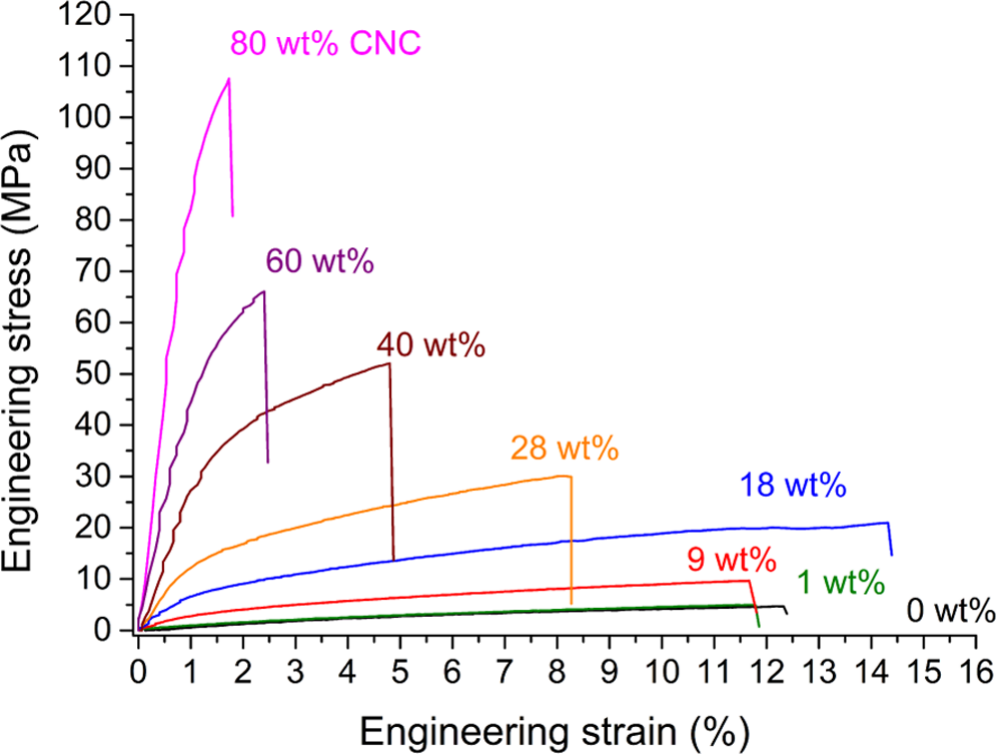
Overlay of representative tensile curves obtained from the UV-cured films of Latex2OA containing various amounts of CNC as a reinforcing filler.

**Figure 9:**
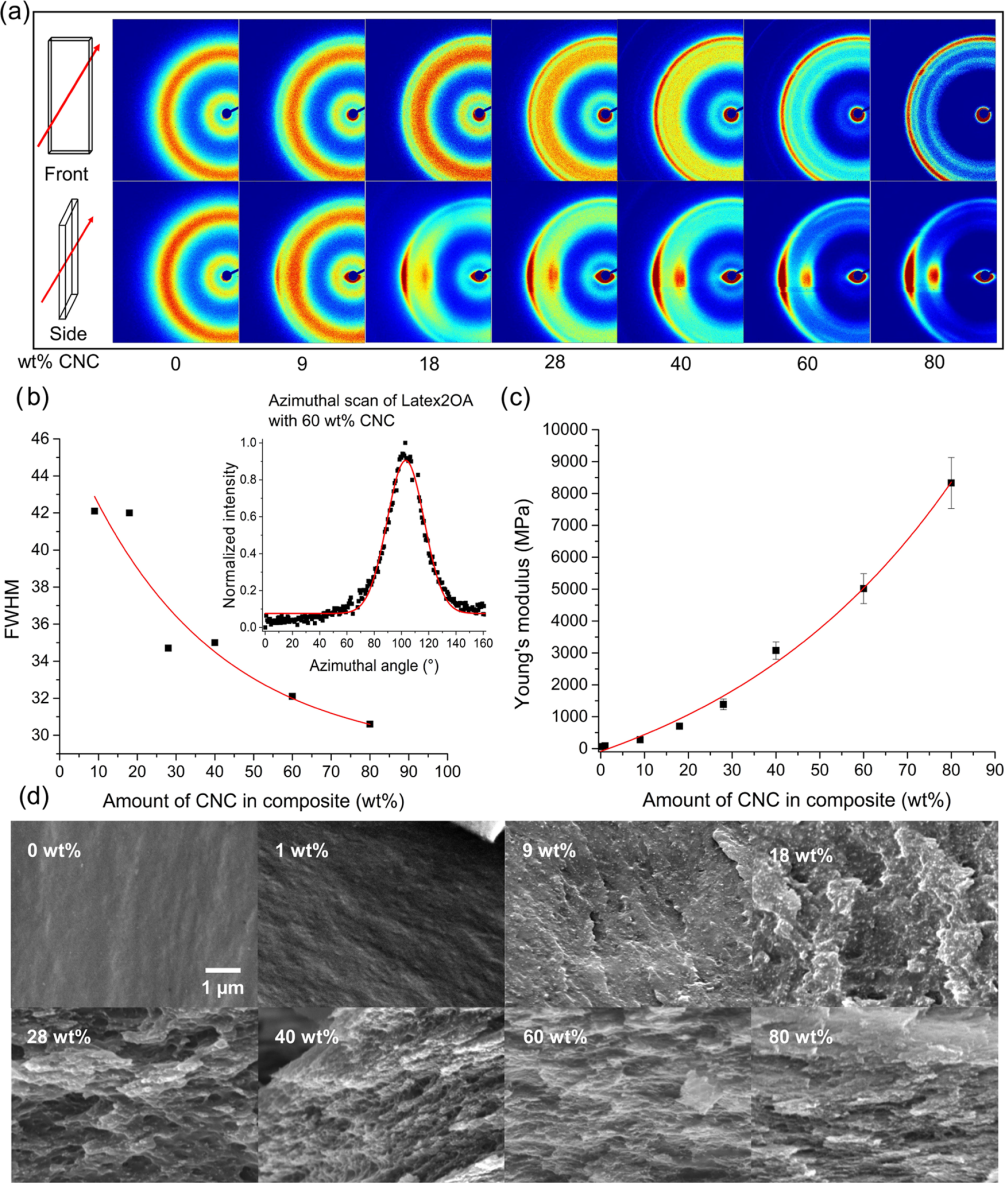
(a) 2D WAXD patterns of the CNC-loaded films from Latex2OA measured from the front and side of the film. The orientation of the film in the sample holder was vertical. (b) Full width at half-maximum (fwhm) of the signal from the azimuthal angle of the 2D WAXD patterns as a function of the amount of CNC in the composite. (c) Young’s modulus of the films as a function of the amount of CNC in the composite. (d) SEM images of the fraction surfaces of the CNC-loaded films with a magnification of 16000 ×. The orientation of the sample is horizontal.

Indication of the reinforcing effect of CNC in the polymer latex films was the mechanical performance observed during tensile testing. Due to the rodlike structure of CNC, improvements in the stiffness are expected. The results in Table S9 display significant improvements in the Young’s modulus for Latex10, 25, and 2OA containing 9 wt % CNC or more. In the case of Latex2OA, a drastic increase in the Young’s modulus was observed with an increasing fraction of CNC in the composite (Figure 8). Overall, the strain at break decreases with increasing fraction of CNC; however, an optimum in mechanical properties is reached with about 18 wt % CNC in the composite yielding the highest strain at break of 16 ± 3.2%. Compatibilization of CNC with polymer matrix was previously achieved with the addition of a hydrophilic polymeric compatibilizer such as poly(vinyl alcohol) or PEG.^42^ In this case, the POEGA macro-RAFT agent fulfills this role. It appears that at 18 wt % CNC, there exists a favorable ratio between CNC, POEGA, and hydrophobic polymer matrix that results in the increases in both tensile strength and strain at break. Further increase of the CNC content up to 80 wt % still resulted in transparent and homogeneous films but reduced the strain at break. Instead, Young’s modulus showed an increase as a function of the amount of CNC in the composite. Composite films from Latex2OA had a Young’s modulus of 63 ± 16 MPa at 0 wt % CNC and 8330 ± 798 MPa at 80 wt % (Figure 9c).

The presence of CNC in the composite was confirmed by FTIR spectroscopy (Figure S13) and WAXD (Figure S14). With the increasing amount of CNC in the composite, the characteristic signals belonging to CNC in the 1D WAXD signals became more prevalent.^43^ 2D WAXD characterization of the CNC-loaded films was performed from two directions on the sample. The X-ray beam was directed on the front of the sample and from the side, which distinguishes in-plane and through-plane orientations of the CNC’s that can occur as a result of the casting and drying process. The 2D WAXD patterns measured from the front show an isotropic signal (Figure 9a). The samples measured from the side clearly show orientation, judging from the localized signal distribution perpendicular to the sample orientation, which was vertical (Figure 9a), suggesting a layered in-plane oriented structure. The full width at half-maximum (fwhm) of the azimuthal signal from the 2D WAXD patterns gives an indication of the relative degree of CNC orientation (Figure 9b). The fwhm decreases with an increasing amount of CNC in the composite, suggesting an increase in orientation. Both increases in the amount of reinforcing filler and increased orientation thereof support the observed relationship between the mechanical properties and amount of CNC in the films (Figure 9c). Further evidence of the orientation of the CNC in the film was supplied by SEM imaging of the fracture surfaces (Figure 9d). Clear transitions were observed from a smooth surface for the 0 and 1 wt % CNC-loaded films to a more coarse and speckled surface for the 9, 18, and 28 wt % CNC-loaded films. No evidence of microscale agglomeration of CNC was observed in the SEM for these samples, suggesting a good distribution of the filler in the matrix. With CNC loadings of 40, 60, and 80 wt %, a transition to a layered and oriented structure is observed. The layers are in-plane oriented (the sample orientation in the images is horizontal) and, therefore, correlate well with the observations made with WAXD. The layered morphology resembles a nacre-like structure, which is known for contributing to high-stiffness materials and suggests promise in barrier applications.^44,45^

**Figure 10:**
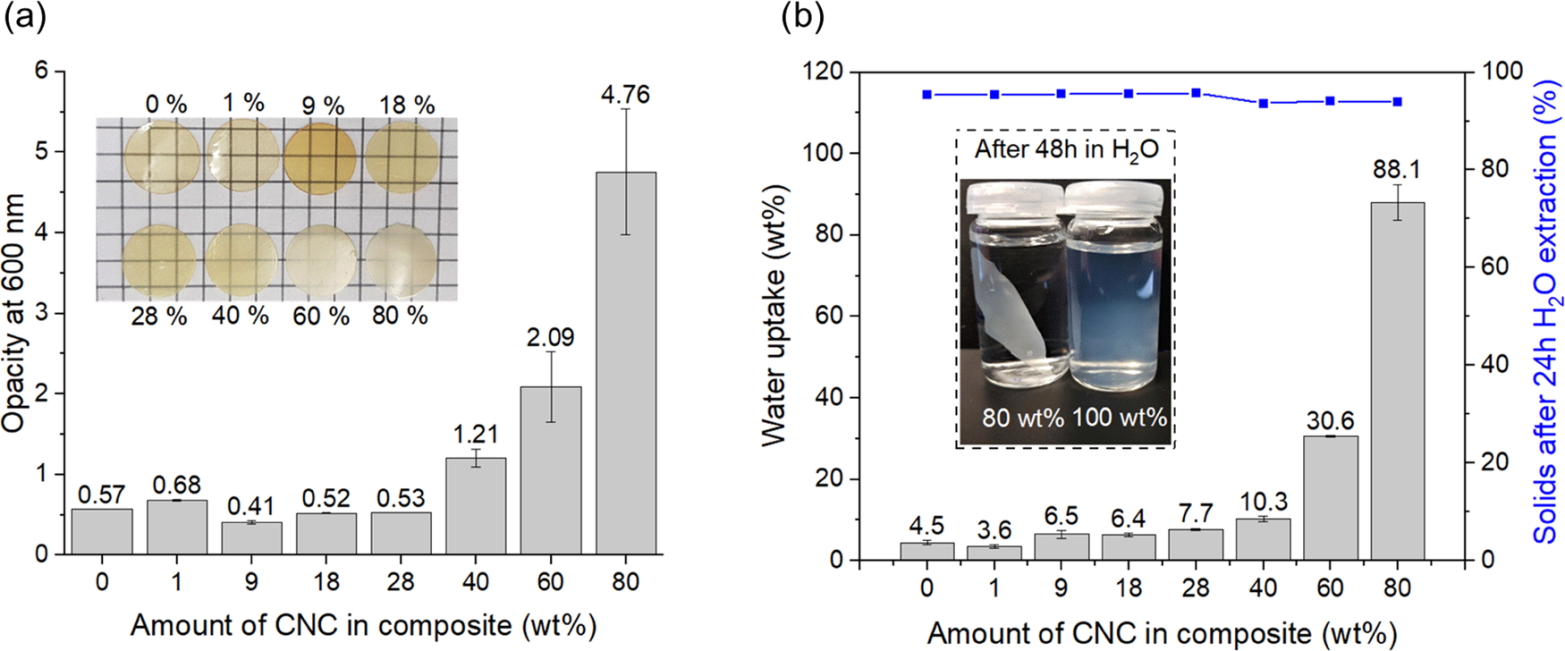
(a) Opacity of the CNC-loaded films measured by UV–vis at a wavelength of 600 nm. (b) Water uptake after submersion for 48 h and solid content after extraction with H_2_O of the CNC-loaded films.

The opacity of the films was measured using UV–vis and remained largely unaffected up to 28 wt % CNC (Figure 10a). While all films were optically transparent and homogeneous, a further increase in the CNC content of 40, 60, and 80 wt % resulted in a significant increase in opacity (Figure 10a). These results correlate with the observations made in SEM (Figure 9d), also indicating a clear morphological transition between 28 and 40 wt % CNC in the composite.

The water uptake of the films was influenced by the amount of CNC in the composite film (Figure 10b). One of the major drawbacks of films composed of CNC is the high water sensitivity due to the disruption of hydrogen bonding at the interface between the cellulose crystals.^46,47^ However, in the case of the present materials, swelling in water was largely negated. After submersion into water for 48 h, all films remained intact and similar in appearance. Even the films containing 80 wt % retained their structure, whereas a film based on pure CNC rapidly disintegrated after introduction to water (Figure 10b). Only slight increases in the water uptake were observed for films containing up to 40 wt % CNC. The films containing 60 and 80 wt % CNC showed a more significant water uptake of 30.6 and 88.1 wt %, respectively. We hypothesize that a a good distribution of the UV-cross-linked hydrophobic latex effectively diminishes the destabilizing effect of water on the CNC’s. All of the films retained more than 94% of their mass after 24 h Soxhlet extraction with both THF and water, while pure CNC leaves no residues after extraction with water. Furthermore, the surface contact angle with water was not decreased as a result of CNC incorporation (Table S10).

## Conclusions

In this work, we have successfully developed the synthesis of renewable latexes based on polymerization-induced self-assembly using a macro-RAFT agent. The innovation is the use of the functional and renewable monomer 4CPA that can undergo postpolymerization UV curing without the addition of an external cross-linker, sensitizer, or initiator. UV-cross-linkable side groups allow for the separation of the film formation and curing step, improving flexibility in the application of this material. Postcuring also overcomes one of the major contradictions in water-borne latexes, which desire both facile film formation at ambient temperature while avoiding the use of solvents and combining this with good hardness, blocking resistance, and solvent resistance.

The latex film formation was investigated in a series of selected latexes with a solid content of between 34.6 and 47.1 wt %, small particle size, good colloidal stability, and low viscosity. Film formation followed by UV curing by photocyclodimerization of the cyclopentenone side groups leads to mechanically rigid films. Since the extent of dimerization, and thus cross-linking, is dependent on the UV exposure time, the properties of the film could be tuned from soft and flexible to rigid. Application of the latex on a substrate leads to films with high hardness, good solvent resistance, and blocking resistance as a result of the UV curing process. Depending on the monomer composition of the latex, the surface contact angle with water is between 64.3 and 96.3°, indicating that the choice of monomer type and ratio yields different surface interactions with water. The UV curing step also drastically increased the contact angle of all evaluated films, suggesting a change in the chemical or morphological nature of the surface.

As a proof of concept, coatings on a paper substrate were prepared in order to evaluate the oil and water barrier properties. A low coating weight of 3.3 g/m^2^ drastically reduced the amount of oil and water absorption by the substrate compared to uncoated paper and formed an effective oil barrier according to the KIT test results. At the same time, the absorbency and gloss of the paper could be further improved by applying thicker layers. As a water and oil barrier layer on paper, the coating performs on a similar level as commercial styrene-acrylate binders that are used in paper coating formulations.

The morphology of the latex particles containing POEGA chains that are covalently attached allows for interaction with hydrophilic fillers that are able to undergo hydrogen bonding. Therefore, cellulose nanocrystals (CNC) were investigated as potential fillers, showing effective improvement of the mechanical properties of the freestanding films. By increasing the amount of CNC from 0 to 80 wt %, the Young’s modulus showed an increase. XRD measurements and SEM imaging confirmed the in-plane layered orientation of the CNC crystals in the polymer film matrix that became more oriented with an increasing amount of CNC. The contribution of the rigidity of the CNC filler and the alignment might explain the relationship between the filler amount and Young’s modulus. Further characterization of the freestanding films showed that the water absorption remains low, retaining the structural integrity after submersion in water. Water absorption and opacity, however, increased with increasing amounts of CNC. The simple mixing of CNC dispersion with the emulsions is a facile and effective strategy to introduce CNC in a hydrophobic matrix and alleviate many of the problems that are associated with CNC films, mainly related to the high water sensitivity. Further research could recognize the implementation of these latex CNC composites as gas, oil, and water barrier films.

## Supporting Information Available

Details on experimental section; results on the development of 4CPA latex synthesis in small-scale experiments; results on the rheological characterization of the latex/CNC mixtures; supplementary tables and graphs; and literature overview of Cobb60 water absorption values, DLS data of latexes, latex stability evaluation, surface active properties of latexes, surface tension data on the POEGA macro-RAFT agent, rheology curves of the latexes, characterization results of the freestanding films, addition results on the tensile properties of freestanding films produced under various conditions, properties of the reference latex, tensile graph of the reference latex, TGA results of the freestanding films, FTIR spectra of the CNC-loaded films, and 1D WAXD data of the CNC-loaded films (PDF)
