# Supplementary material for: Renewable and Functional Latexes Synthesized by Polymerization-Induced Self-Assembly for UV-Curable Films
Source: ACS Appl Mater Interfaces. 2023 Nov 6;15(45):52939–52. doi: 10.1021/acsami.3c11657 (PMC10658448; doi:10.1021/acsami.3c11657)
Supplement: Supplementary file 1 — am3c11657_si_001.pdf [file am3c11657_si_001.pdf]

Supporting Information

Renewable and Functional Latexes Synthesized by Polymerization Induced Self-Assembly for UV Curable Films

Jules Stouten[a], Huixing Cao[a], Andrij Pich[a,b], Katrien V. Bernaerts[a]*

[a] Aachen-Maastricht Institute for Biobased Materials (AMIBM), Faculty of Science and Engineering, Maastricht University, Brightlands Chemelot campus, Urmonderbaan 22, 6167 RD Geleen, the Netherlands.

[b] DWI Leibniz-Institute for Interactive Materials, Aachen 52056, Germany; Institute of Technical and Macromolecular Chemistry (ITMC), RWTH Aachen University, Aachen 52074, Germany

* To whom correspondence should be addressed.

List of supplementary information:

Section 1: Experimental section details and characterization

Section 2: Development of 4CPA latex synthesis

Section 3: Rheological characterization of the latex / CNC mixtures

Section 4: Supplementary tables and graphs

1. Experimental section details and characterization

Table S1. Characterization results of the POEGA macro-RAFT agent.

Polymer	Molar ratio	OEGA conversion	$M_{n, th}$ [a]	$M_{n, NMR}$ [b]	$M_{n, GPC}$	Đ	Yield
	I:RAFT:OEGA	(%)	(kg/mol)	(kg/mol)	(kg/mol)		(g)
POEGA macro-RAFT	0.07:1:10	78	4.1	4.5	5.9	1.06	29.7 (70%)

[a] Calculated as follows: Target DP OEGA × Conversion OEGA × 480 g/mol + 318 g/mol / 1000.

[b] Calculated from the ^{1}H NMR spectrum in Figure S1: Integral of resonance f / Integral of resonance h × 480 (g/mol) + 318 (g/mol) / 1000.

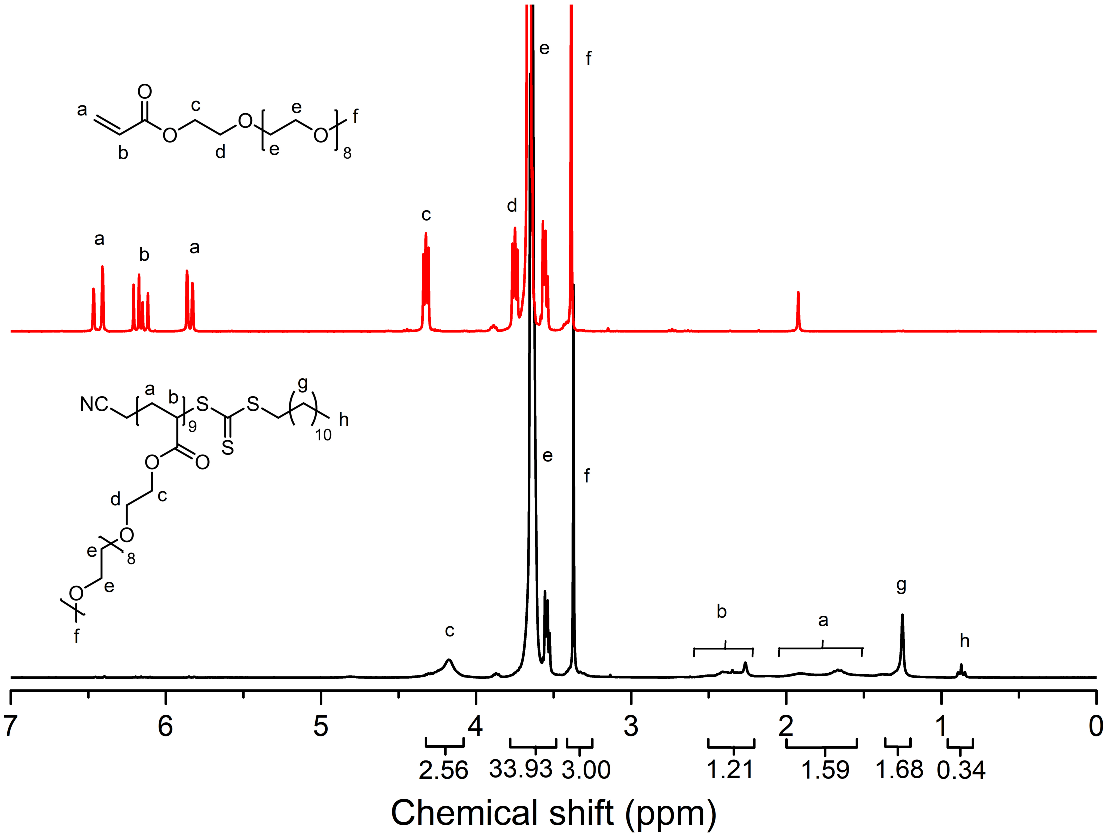

Figure S1. ^{1}H NMR spectrum overlay of OEGA monomer and POEGA macro-RAFT agent in $CDCl_3$.

Characterization

Gel Permeation Chromatography (GPC)

Gel permeation chromatography (GPC) was performed at 30 °C using a Waters GPC equipped with a Waters 2414 refractive index detector. Tetrahydrofuran (THF) was used as the eluent at a flow rate of 1 mL/min. Three linear columns (Styragel HR1, Styragel HR4, and Styragel HR5) including a Styragel Guard column were used. Molecular masses are given relative to polystyrene standards. The polymers were dissolved in THF with a concentration of 3 mg/mL and filtered over a 0.2 μm PTFE syringe filter.

Nuclear Magnetic Resonance (NMR) spectroscopy

^{1}H NMR (300 MHz) spectra were recorded on a Bruker Avance III HD Nanobay 300 MHz apparatus at 298K in $CDCl_3$ using 16 scans. NMR spectroscopy was used for polymer structural confirmation and for the determination of monomer conversion in the macro-RAFT agent synthesis. The disappearance of the vinyl resonances relative to the trioxane (internal standard) resonance were followed over time. Prior to NMR measurement, the latex was dried in a vacuum oven at 40 °C for 24 hours.

Gas Chromatography with Flame Ionization Detector (GC-FID)

Individual monomer conversions during emulsion polymerization were determined using gas chromatography with flame ionization detection (GC-FID). Aliquots taken from reaction mixtures were dissolved in THF prior to measurement. The disappearance of the monomer peaks relative to naphthalene (internal standard) were followed. Measurements were performed on a Shimadzu GC-2010 equipped with a Supelco SPB-1 capillary column (30 m × 0.25 mm × 0.25 μm film thickness). The temperature program was as follows: an initial temperature of 80 °C was maintained for 3 min and then increased to 140 °C with a heating rate of 10 °C/min. This temperature was maintained for 1 min and further increased to 300 °C with a heating rate of 20 °C/min and was maintained at 300 °C for 5 min (the total run time of 23 min).

Differential Scanning Calorimetry (DSC)

The glass transition temperature (T_g) of polymer samples were determined using a Netzsch DSC 214 Polyma instrument. Prior to measurement, the samples were dried at 40 °C in a vacuum oven. The samples were heated in 2 cycles from -40 °C to 100 °C or from -60 °C to 100 °C with a rate of 10 °C/min under nitrogen atmosphere. The second cycle was used for determination of the phase transition points. The inflection point was used for reporting of the T_g.

Thermogravimetric analysis (TGA)

TGA on dried polymer films was performed on a TA Instruments TGA Q500 under nitrogen atmosphere. The samples were heated from 25 °C to 700 °C with a rate of 10 °C/min.

Dynamic Light Scattering (DLS)

The particle size distribution of the 4CPA latexes was determined using DLS. The aqueous emulsions were diluted with distilled water until they became visually slightly turbid. The samples were measured on a Malvern Instruments Zetasizer Nano ZS DLS instrument at 25 °C and a fixed angle of 173°. Zeta potential measurements were performed with the same instrument, using folded capillary cells.

Wide Angle X-ray Diffraction (WAXD)

The presence and orientation of CNC in the polymer was assessed with 2D wide-angle X-ray diffraction. 2D WAXD patterns were recorded on a SAXSLAB Ganesha instrument using Cu Kα radiation ($\lambda = 0.154$ nm). The beam center and θ-range were calibrated using the diffraction pattern of silver behenate. The conversion of 2D into 1D data was performed using the Saxsgui v2.13.01 software.

Cryogenic Electron Microscopy (Cryo-TEM)

The 4CPA latexes were visualized by the Cryo-TEM method. The latex was firstly diluted to 1 wt% with distilled water. A thin aqueous film was formed by applying a 5 µl droplet of the suspension to a bare specimen grid. Glow-discharged holey carbon grids were used. After the application of the suspension, the grid was blotted against filter paper, leaving thin sample film spanning the grid holes. These films were vitrified by plunging the grid into ethane, which was kept at its melting point by liquid nitrogen, using a Vitrobot (Thermo Fisher Scientific Company,

Eindhoven, Netherlands) and keeping the sample before freezing at 95% humidity. The vitreous sample films were transferred to a Tecnai Arctica cryo-electron microscope (Thermo Fisher Scientific, Eindhoven, Netherlands). Images were taken at 200 kV with a field emission gun using a Falcon III direct electron detector.

Scanning Electron Microscopy (SEM)

SEM was performed on a Jeol JSM-IT200 scanning electron microscope operated at 10 kV. Sample preparation was performed by freezing the sample in liquid nitrogen before fracturing the specimen in half. The sample was mounted with the fracture surface facing upward. The samples were sputtered with gold under argon atmosphere.

Gel content determination

Of every latex, several droplets were deposited on a piece of accurately weighed cellulose filtration paper (W_1). The filter paper was dried overnight and weighed (W_2). Subsequently, the sample was extracted with THF in a Soxhlet extractor for 24 hours. Afterwards, the sample was dried and weighed (W_3). The gel content was calculated as follows:

Eq. 1 $$Gel\ content\ (wt\%) = \frac{(W_3 - W_1)}{(W_2 - W_1)} \times 100$$

Weighing of the sample and filtration paper was performed on an automatic drying balance, which heated the sample to 105 °C until a steady weight was obtained.

The gel content of cross-linked films was performed by extracting an accurately weighed piece of film (W_1) with THF or water in a Soxhlet extractor for 24 hours. After extraction, the films were dried in a 40 °C vacuum oven overnight and weighed again (W_2). The gel content was calculated as follows:Eq. 2 $$Gel\ content\ (wt\%) = \frac{W_2}{W_1} \times 100$$

Solid content

The solid content of the latexes was measured by accurately weighing an amount of latex in an aluminum dish (W_1). The latex was dried in a 60 °C vacuum oven for 24 hours after which the solids were weighed (W_2). The solid content was calculated as follows:

Eq. 3 $$Solid\ content\ (\%) = \frac{W_2}{W_1} \times 100$$

Latex stability

The stability of the latexes was evaluated by the freeze-thaw method and by the addition of salt solution and solvent (ethanol). For the freeze-thaw evaluation, 1 mL of latex sample was frozen for 24h at -20 °C and then thawed at room temperature. For the evaluation of addition of salt solution and solvent, 1 equivalent of salt solution or solvent was added to 1 mL of latex sample. The latex was visually inspected for phase separation prior and after each latex stability method. Also, a DLS measurement was performed to evaluate the effect on the particle size.

Solvent swelling ratio

The swelling ratio of cross-linked polymer films was determined by placing an accurately weighed piece of film (W_1) (about 100 – 200 mg) in a flask containing 10 mL THF for 24 hours. The film was taken out of the flask and excess solvent on the surface was removed before weighing (W_2). The swelling ratio was calculated as follows:

Eq. 4
$$Swell\ ratio = \frac{(W_2 - W_1)}{W_1}$$

Water uptake

The water uptake of the cross-linked polymer films was determined by placing an accurately weighed piece of film (W_1) (about 100-200 mg) in a flask containing 10 mL distilled water for 48 hours. The film was taken out of the flask and excess solvent on the surface was removed before weighing (W_2). The water uptake was calculated as follows:

Eq. 5
$$Water\ uptake\ (\%) = \frac{(W_2 - W_1)}{W_1} \times 100$$

The water uptake tests were performed three times on each sample. The reported values are the average and the reported errors are standard deviations between three tests.

Minimum Film Formation Temperature (MFFT)

The MFFT was measured on a Rhopoint MFFT Bar 90 instrument according to ASTM D 2354.

Tensile test

The mechanical performance of the freestanding polymer films was assessed by tensile testing. Tensile tests were performed on a Linkam micro tensile stage equipped with a 200N or 20N load

cell. Freestanding films with a thickness of about 0.2 mm were cut into dog bone shaped specimens with dimensions of 31 × 2 mm (length × width), the broad part of the dog bone specimen had a width of 4 mm. The tensile tests were performed with a deformation rate of 33.3 μm/s. All measurements were performed at room temperature. The Young's modulus of each measurement was determined between 0.2 and 0.6% strain. The values reported are the average of five tensile measurements and errors reported are the standard deviations.

Rheology

Rheological measurements were performed on a TA Instruments DHR rheometer equipped with a 40 mm parallel plate and Peltier element. Pure latexes and mixtures with CNC dispersion were deposited on the Peltier element controlled at 25 °C and the gap was set at 1000 μm. Frequency sweep experiments were performed at 2% deformation. Steady shear and oscillatory experiments were carried out to investigate the effect of shear rate on viscosity and viscoelastic properties. The hysteresis loop (performed with step times between 30 s and 600 s) test and the three interval thixotropy test (3ITT) (alternating between low shear 1 s^{-1} for 90 s, and high shear 100 s^{-1} for 120 s) were carried out to investigate the thixotropic behavior. Flow sweep measurements were carried out with a shear rate from 0.01 to 1000 s^{-1}.

Raman spectroscopy

Raman spectra were recorded on a Bruker RFS 100/S Raman spectrophotometer between 400 and 3500 cm^{-1}, using a resolution of 4 cm^{-1} and 700 scans. The sample was ground into a powder prior to measurement.

UV-Vis spectroscopy

The opacity of CNC loaded films was measured using UV-Vis spectroscopy. Measurements were performed on a Shimadzu UV-3600 UV-Vis-NIR spectrophotometer. The spectra were recorded from 700 to 200 nm with a slit width of 32 nm. The transmittance at 600 nm of each film was recorded and the opacity was calculated according to the following equation.

Eq. 6 $$Opacity = -\log(T600)/x$$

Where T600 is the fractional transmittance at a wavelength of 600 nm, and x is the film thickness in mm. A lower transmittance results in a higher opacity value.

Fourier Transform Infrared (FTIR) spectroscopy

Attenuated Total Reflection FTIR spectroscopy (ATR-FTIR) was performed on a PerkinElmer Spotlight 400 equipped with a PIKE GladiATR. Accumulations of 64 spectra were collected in the range of 4000−400 cm^{-1} with a spectral resolution of 2 cm^{-1}.

Film characterization techniques

Layer thickness

The dry layer thickness of the cured coatings was determined with an Elcometer 456 layer thickness device by taking the average of the layer thickness at five different spots.

Blocking resistance

The blocking resistance of polymer films was determined according to the following procedure. Films on Leneta cards were cut in strips of 5 cm × 3 cm and stacked face to face. The strips were loaded with a pressure of 1 Kg for 4 hours at 50 °C. The visual evaluation of the films was performed according to standard method ASTM D 2793-99. Grades were appointed to each film evaluating the degree of blocking and the surface damage according to the following criteria. Grades were appointed to each coating evaluating the degree of blocking (from A = free fall separation to F = tool required to separate) and the surface damage (from 0 = none to 5 = >50% damage).

Contact angle

Contact angle measurements were performed on an Attension Theta optical tensiometer. Using the equipment sample stage, a drop of distilled water (5 μL) was deposited on the film surface in front of a camera. After 10 seconds equilibration time, the angle between the film surface and tangent line of the water droplet was measured using the OneAttension analysis software. The reported values are the average of five measurements. The reported errors are the standard deviations.

Surface tension

The surface tension was determined by the pendant drop method using an Attension Theta optical tensiometer. Block copolymer solutions with known concentration were prepared in milli-Q water. The surface tension was calculated by the OneAttension analysis software from the shape of the

drop (5 μL) using the Young-Laplace equation. The concentration range that was evaluated was between 5×10^{-5} and 2.1×10^{-2} mM. The critical aggregation concentration (CAC) was calculated at the intersection of the tangent lines of the linear region and the plateau.

Water and Methyl Ethyl Ketone (MEK) double rub

The water and solvent resistance was evaluated by the double rub method. Films on steel plates were gently rubbed with a cotton swab that was previously immersed in distilled water or MEK. The amount of double rubs (one time back and forth) were counted for each test until visual damage on the surface appeared. The double rub tests were performed three times on each sample. The reported values are the average and the reported errors are standard deviations.

Gloss

Gloss measurements were performed on films applied on steel substrates using a BYK micro-TRI-gloss instrument at 20°, 60°, and 85°. The average of three measurements at different spots was taken as the final value.

Cross-cut adhesion

Adhesion of the films applied on steel substrates was evaluated using an Elcometer 1542 cross-cut adhesion tester. The adhesion test and evaluation was performed according to ASTM D 3359-B. After the test, the tested area was classified into grades between 5B for no surface damage, and 0B for 65% to complete flaking.

Pencil hardness

The hardness of coated films applied on steel substrates was evaluated using an Elcometer 3086 motorized pencil hardness tester according to ASTM D 3363. The hardness was evaluated using a range of leads between 6H and 6B. A weight of 7.5 N on the lead was used. The hardest lead, which did not leave a scratch on the film surface, was selected as the hardness value.

KIT grease resistance test

The KIT grease resistance test was performed in accordance with the standard Tappi 559 pm-96. The mixtures of castor oil, toluene and n-heptane were prepared according to Table S2. A drop of the test liquid was deposited on the film and wiped after 15 seconds. The darkening as result of

wetting of the oil on the underlying substrate was visually examined. If darkening occurred, the test failed and a lower KIT no. was tested. The highest number that does not cause darkening is reported. A higher KIT value indicates better oil barrier properties. The test was repeated three times on three different coatings. The values were averaged to the nearest 0.5.

Table S2. Composition of the mixtures used in the KIT grease resistance test.

KIT no.	Castor oil (mL)	Toluene (mL)	n-Heptane (mL)
1	100	0	0
2	90	5	5
3	80	10	10
4	70	15	15
5	60	20	20
6	50	25	25
7	40	30	30
8	30	35	35
9	20	40	40
10	10	45	45
11	0	50	50
12	0	45	55

The films that were evaluated were prepared by applying a layer of latex with a wet film thickness of 30, 90, or 120 μm using a manual bar coater on bleached uncoated paper with an average weight of 78 ± 1 g/m^2. The film was dried for several hours before UV curing for 40 minutes in a nitrogen filled Dymax UV chamber equipped with a 400 W metal halide UVA lamp.

Cobb-Unger oil and water absorbency

The Cobb-Unger oil and water absorbency test was based on Scan-P 37:77 procedure. The absorbency value in g/m^2 was calculated according to the following equation.

Eq. 7 $$Absorbency = \frac{(W_2 - W_1)}{A}$$

Where W_1 is the weight of the sample before exposure to liquid, and W_2 is the weight after exposure to the liquid. A indicates the area of the test specimen, which was 0.001452 m^2. The evaluated liquids were castor oil and water. The test specimens were exposed to castor oil for 10 minutes and water for 60 seconds. In short, the test was executed by filling a cup with the test

liquid followed by placing the test specimen on the cup. When the cup was turned upside down, a timer was started. Five seconds before the end of the exposure, the cup was turned and excess liquid was blotted using paper tissue. The average absorbency value was taken over three measurements.

2. Development of 4CPA latex synthesis

Firstly, we explored the reactivity of 4CPA with comonomers in solution by determining the reactivity ratios via the method described by Jaacks.[1] Previous research has shown that 4CPA shows a slightly higher reactivity in the RAFT controlled copolymerization with several acrylate comonomers.[2] This is the case for IBOA, THFA, and 2OA, where the reactivity ratio of 4CPA is >1 and the comonomer reactivity ratio is <1 in all monomer pairs (Figure S2c and Figure S3). Nevertheless, $r_1 \times r_2$ is close to 1 in all cases suggesting a near statistical copolymerization. This suggests that the observed differences in monomer reactivity reside in different transport rates through water in the heterogeneous systems. This is especially true in the early stages of the emulsion polymerization of Latex2OA (Figure S2b), where 4CPA shows reactivity but the comonomers do not.

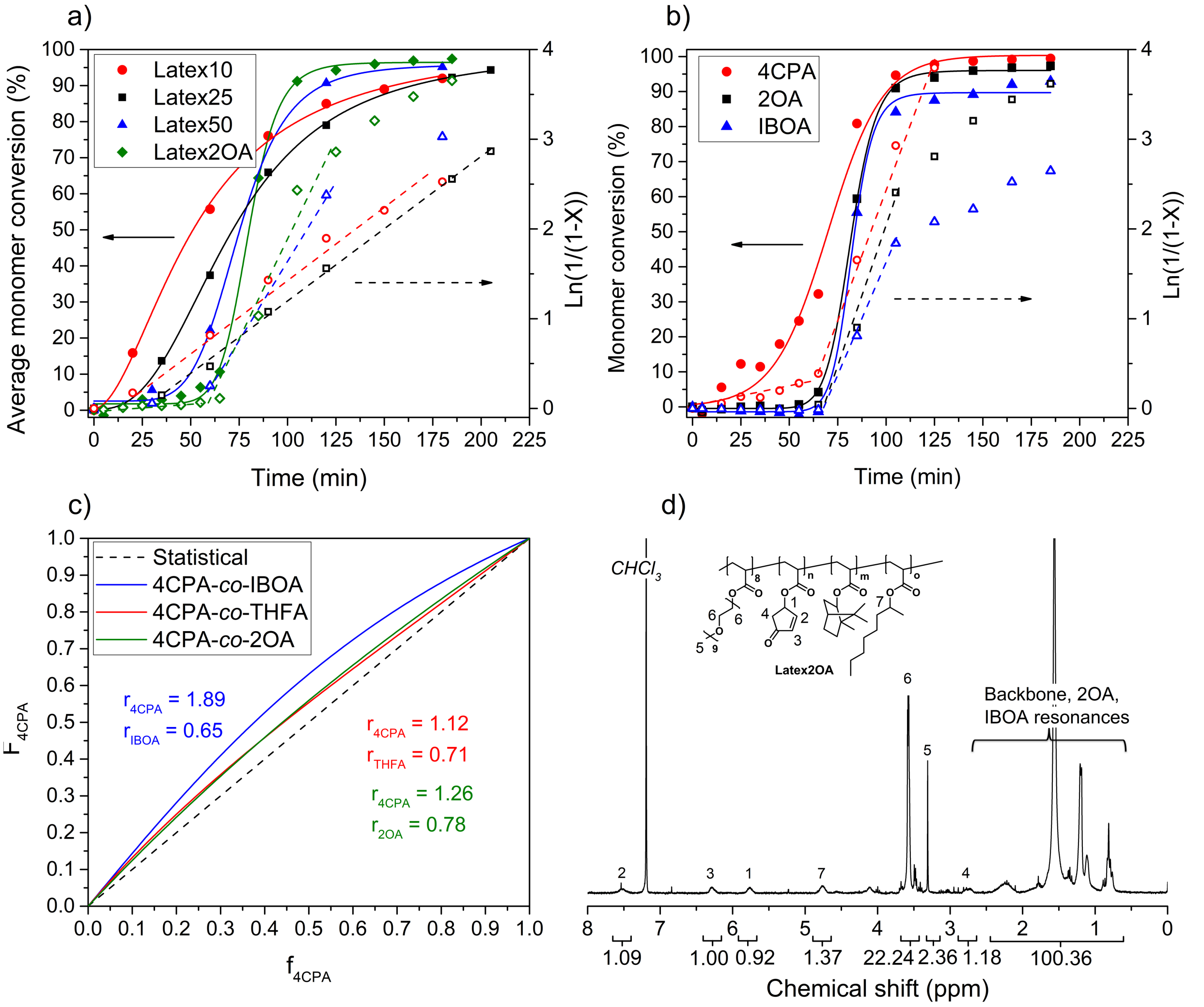

Figure S2. Copolymerization of 4CPA a) average monomer conversion as a function of time and first order plots of latexes 10, 25, 50, and 2OA. b) Individual monomer conversion and first order plots in the emulsion polymerization of Latex2OA, c) Copolymerization graph of 4CPA with comonomers IBOA, THFA, and 2OA. d) ^{1}H NMR spectrum of dried Latex2OA.

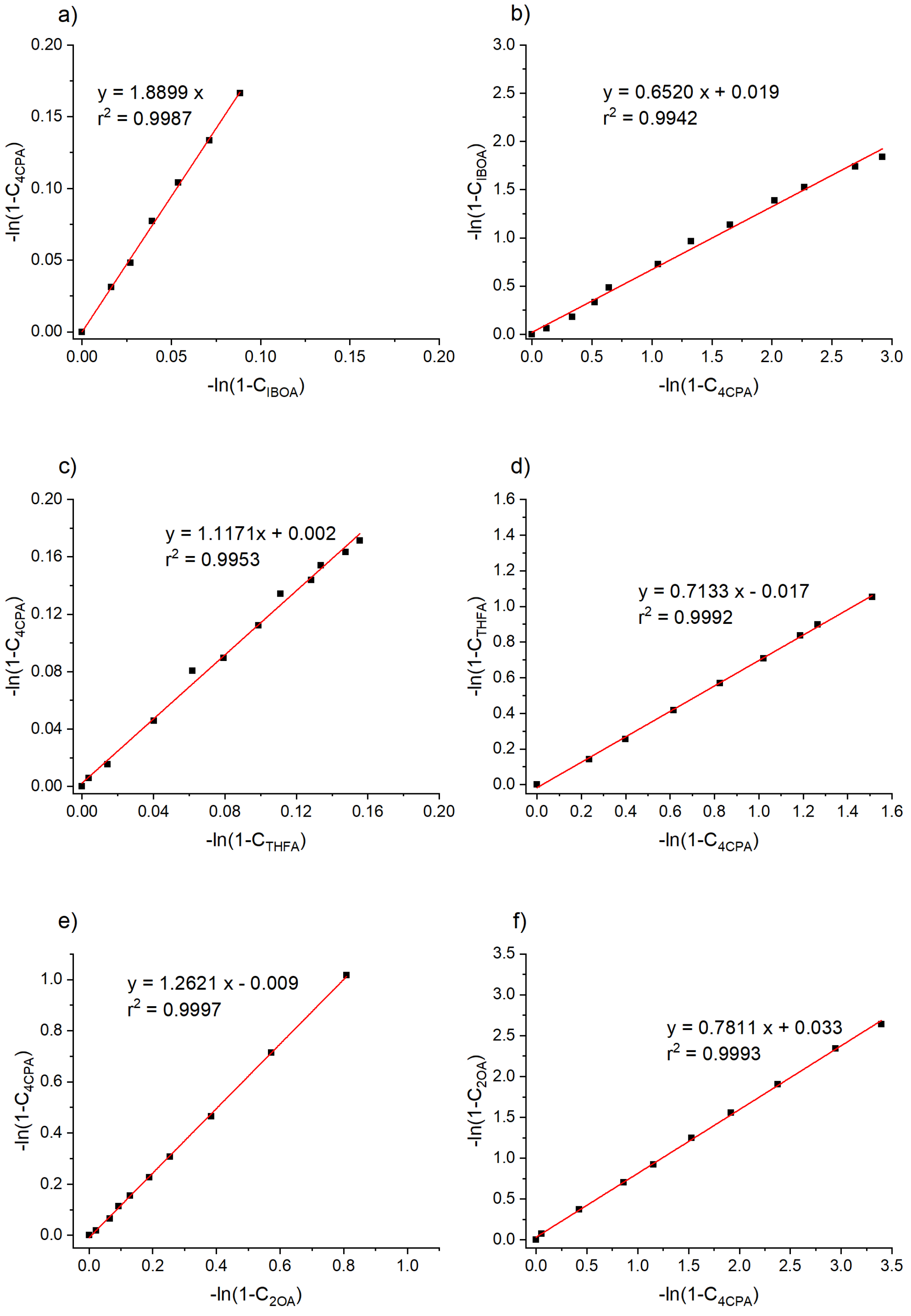

Figure S3. Jaacks plots of the copolymerization of 4CPA with comonomers IBOA, THFA, and 2OA. a) Excess 4CPA relative to IBOA. b) Excess IBOA. c) Excess 4CPA relative to THFA. d) Excess THFA. e) Excess 4CPA relative to 2OA. f) Excess 2OA.

Firstly, several emulsion polymerization reactions without 4CPA in the monomer feed were investigated using the POEGA macro-RAFT agent at 50 °C. Despite the high LCST temperature of $POEGA_{480}$ of 92 °C[3] we found that performing the RAFT PISA at 50 °C in contrast to 65 °C, allowed for much higher solid content values while in the meantime the particle size decreased. The reactions containing various amounts of butyl acrylate (BA), THFA, and IBOA in the feed served to test if controlled polymerization conditions are maintained using the POEGA macro-RAFT agent. Furthermore, the reactions should lead to near complete monomer conversion, and yield a stable emulsion with small particle size. The results are summarized in Table S3. All reactions resulted in high monomer conversion of >96%.

In Figure S4a, the monomer conversion is plotted as a function of time. The monomer conversion can be facilely followed using GC-FID revealing the extent of polymerization. The characteristic S shaped curve is formed by an initial lag phase, which is followed by a rapid increase in the monomer conversion. This transition marks the formation of self-assembled particles in which polymerization continues through the fast transport of monomer to the particles. It is at this point that also a strong visual change of the reaction mixture takes place, from semi-transparent to completely turbid and milky white with a blue haze. The same phenomenon is observed in the first order conversion plot, which is separated in two linear phases. The first phase marks a slow solution polymerization, while the second phase shows an accelerated reaction rate.

The molecular weight increased linearly with the average monomer conversion indicating controlled polymerization conditions (Figure S4b). The four trials resulted in polymers with a M_n of between 22.8 and 39.9 kg/mol, and a Đ of between 1.14 and 1.81. The emulsions were stable and particle sizes of between 72 and 194 nm were obtained.

Table S3. Results of the small-scale emulsion polymerization without 4CPA using VA-044 as the initiator at 50 °C and performed at 30 wt% solids unless stated otherwise.

	Molar ratio	Monomer feed ratio				Conversion							
Entry	I:RAFT:M	THFA	BA	IBOA	Time (min)	THFA (%)	BA (%)	IBOA (%)	Size (nm)	PDI	$M_{n,th}$[a] (kg/mol)	$M_{n,GPC}$ (kg/mol)	Đ
1	0.5:1:200	0	0.5	0.5	120	-	99	99	92	0.065	37.5	38.7	1.14
2[b]	0.5:1:200	1	0	0	180	98	-	-	194	0.095	34.8	30.4	1.81
3	0.1:1:150	0.5	0	0.5	150	99	-	96	72	0.205	30.8	22.8	1.33
4	0.2:1:300	0.5	0	0.5	150	98	-	98	133	0.051	57.7	39.9	1.62
5[b]	1:1:100	0	1	0	45	-	99	-	45	0.030	16.8	16.7	1.33

[a] Calculated as follows: target DP_{THFA} × $conversion_{THFA}$ × 156.18 + target DP_{BA} × $conversion_{BA}$ × 128.17 + target DP_{IBOA} × $conversion_{IBOA}$ × 208.30 + 4157. [b] Performed at 10 wt% solids. In the calculation of the solid content is considered the amount of monomers in water.

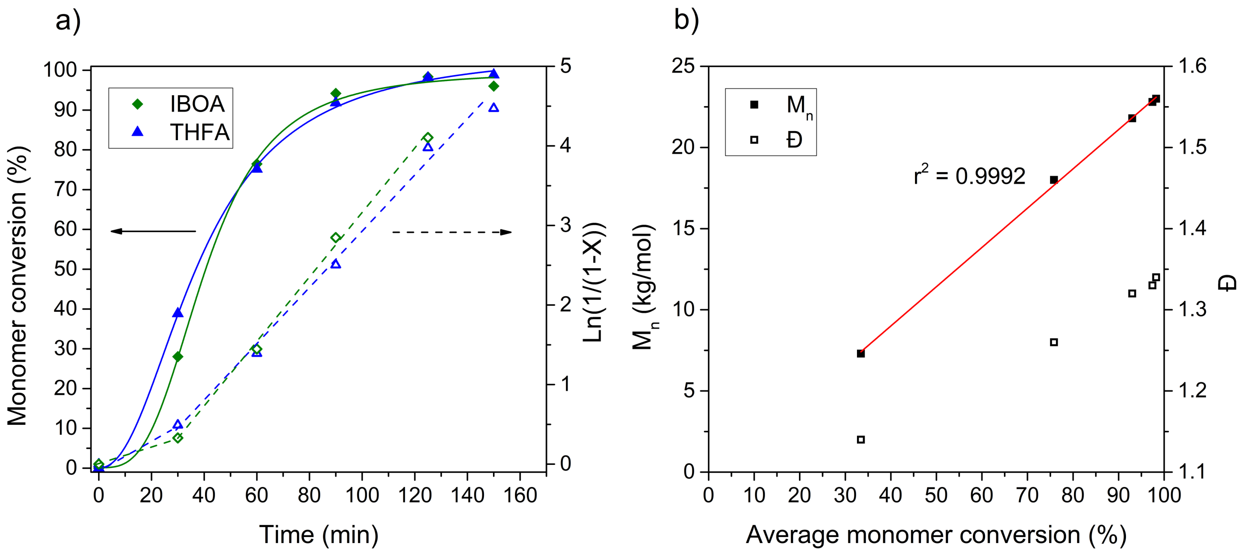

Figure S4. Results of small-scale emulsion polymerization experiment from Table S3, entry 3. a) Monomer conversion as a function of time. b) M_n and Đ as a function of the average monomer conversion.

The next step was the introduction of 4CPA in the monomer feed. In all cases, slight gel formation was observed due to participation of the cyclopentenone unit in the radical polymerization resulting in intraparticle cross-linking. In the experiment series presented in Table S4 is investigated the effect of initiator concentration and solid content on the monomer conversion and particle size. With the addition of 4CPA in the monomer feed, relative large amount of initiator is required to achieve full conversion, which was observed previously in the copolymerization of

4CPA.[2] Nearly complete monomer conversion was obtained with an initiator-to-monomer ratio of 0.5:200. To fully polymerize the remaining monomers de initiator amount can be doubled. Increasing the solid content from 10 to 40 wt% does not significantly affect the monomer conversion. The particle size was increased slightly from 78 to 96 nm, respectively.

Table S4. Optimization of the small-scale emulsion polymerization conditions containing 4CPA, BA, and IBOA in a monomer feed ratio of 0.25:0.40:0.35.

				Conversion					
	**I:RAFT:M**	**Time**	**Solids**	**4CPA**	**BA**	**IBOA**	**Size**	**PDI**	**Gel content**
**Entry**		**(min)**	**(wt%)**	**(%)**	**(%)**	**(%)**	**(nm)**		**(wt%)**
1	1:1:200	180	20	99	99	98	81	0.067	94
2	0.5:1:200	180	20	94	95	93	83	0.03	80
3	0.25:1:200	300	20	18	7	0	N/A	N/A	N/A
4	0.5:1:200	210	10	88	96	96	78	0.033	89
5	0.5:1:200	190	30	90	95	93	89	0.032	86
6	0.5:1:200	150	40	90	87	85	96	0.033	86

To improve the renewable content of the polymer, THFA was investigated as a comonomer to replace BA, which is an established 'soft monomer' in emulsion polymerization. Completely substituting BA and IBOA with THFA resulted in a latex with large particle size of 399 nm and low solid content of 10 wt%. With the introduction of 10 wt% of the more hydrophobic and rigid IBOA, a solid content of 30 wt% was reached while maintaining the particle size of 406 nm. The latex was named Latex10. Further incorporation of IBOA to 25 and 50 mol% resulted in latexes with smaller particle size and higher solid content, named Latex 25 and Latex50, respectively (Table S5). The same latex polymers, but synthesized at a lower solid content of 30 wt% instead of 40 wt%, resulted in a slightly lower particle size. In an additional experiment and under the same conditions, THFA was replaced with 2-octyl acrylate (2OA), another promising biobased monomer for acrylate latexes (Latex2OA, Table S5).

Table S5. Results of the small-scale emulsion polymerization reactions replacing BA with THFA using an initiator-to-RAFT-to-monomer ratio of 1:1:200.

				Conversion						
	**THFA:4CPA:IBOA:2OA**	**Time**	**Solids**	**THFA**	**4CPA**	**IBOA**	**2OA**	**Size**	**PDI**	**Gel content**
**Entry**		**(min)**	**(wt%)**	**(%)**	**(%)**	**(%)**	**(%)**	**(nm)**		**(wt%)**
Latex0[a]	75:25:0:0	240	10	82	89	-	-	399	0.421	89
Latex10	65:25:10:0	150	30	88	93	87	-	406	0.254	76
Latex25_30wt%	50:25:25:0	205	30	93	96	94	-	122	0.56	86
Latex50_30wt%	25:25:50:0	180	30	96	98	93	-	92	0.072	86
Latex25	50:25:25:0	205	40	94	97	93	-	138	0.03	87
Latex50	25:25:50:0	240	40	97	99	93	-	98	0.028	85
Latex2OA	0:25:10:65	190	40	-	99	97	98	100	0.107	93

[a] This experiment was also performed at 30 wt% solids but the mixture gelled.

In general, high monomer conversions were achieved in the polymerizations with THFA and 2OA. In Figure S2a, the typical s-curve of monomer conversion is observed more pronounced in the polymerizations containing the hydrophobic monomers 2OA and IBOA, which experience a longer induction phase. The monomer conversions were followed using GC-FID revealing the extent of polymerization for each individual monomer in the system. The monomer conversion graph of Latex2OA in Figure S2b shows that the more polar 4CPA tends to polymerize in the beginning of the polymerization soon followed by a rapid increase in the monomer conversion of IBOA and 2OA.

Despite the relatively high gel content of the synthesized latexes, ^{1}H NMR spectroscopy of the dried Latex2OA revealed the resonances belonging to all the monomers in the feed (Figure S2d). The resonances at 8.51, 6.29, 5.77, and 2.73 ppm suggest the presence of intact cyclopentenone double bonds, which is required for post-polymerization, UV induced dimerization to obtain dried cross-linked films. Quantification of the double bonds via NMR spectroscopy is not possible due to the partial cross-linking of the latex, visualizing only the soluble fraction of the latex polymer in the ^{1}H NMR spectrum. Therefore, ^{1}H NMR characterization might not reflect the true composition of the latex polymer.

3. Rheological characterization of the latex/CNC mixtures

Two experiments were performed to confirm thixotropic behavior, the hysteresis loop and the three interval thixotropy test (3ITT). The rheology curves belonging to Latex2OA are presented in Figure 7. Flow sweep measurements of the latex / CNC mixtures are presented in Figure S5. In the 3ITT the material is subjected to periods of low (1 s^{-1}) and high (100 s^{-1}) shear rates. As a result of the sudden change in shear rate, the hydrogen bonded network is disturbed and a drastic drop on the viscosity is observed (Figure 7a). The decrease in viscosity is so strong, that almost the same low viscosity of the pure latex is reached at 100 s^{-1}. A sudden decrease in the shear rate resulted in a delayed increase of the viscosity, caused by the build up of the hydrogen bonded network. The 3ITT curves corresponding to Latex2OA, 10 and 25 containing various amounts of 3 wt% CNC dispersion are presented in Figure S6. The difference in viscosity $\Delta\eta_e$ between $\eta_e(1\ s^{-1})$ and $\eta_e(100\ s^{-1})$ depends on the mixing ratio of latex and CNC dispersion. The $\Delta\eta_e$ is especially high for the latexes containing 9 and 18 wt% CNC relative to the solids (Table S6). In most cases, the original viscosity was regained after decreasing the shear rate to 1 s^{-1} within 90 seconds. The exceptions are the mixtures containing 18 and 28 wt%, which showed a recovery in the viscosity of 78 and 76%, respectively. The thixotropic behavior was also visualized by the hysteresis loop, which is formed after linearly increasing and decreasing the shear rate within a certain timeframe.[4] Firstly, the step time was varied from 30 to 600 seconds, which resulted in a larger hysteresis effect with decreasing step time for Latex2OA containing 9 wt% CNC (Figure S7). The hysteresis loop curves of the other latex-CNC mixtures were measured with a step time of 60 seconds (Figure S8). In Figure 7b, the hysteresis loop curve of the Latex2OA containing 18 wt% CNC is shown. The changes in viscosity as a result of shear rate and step time are considerable larger than for the pure latex. Similar behaviour was observed for Latex10, 25, and 2OA containing different amounts of CNC relative to the solids (Figure S8).

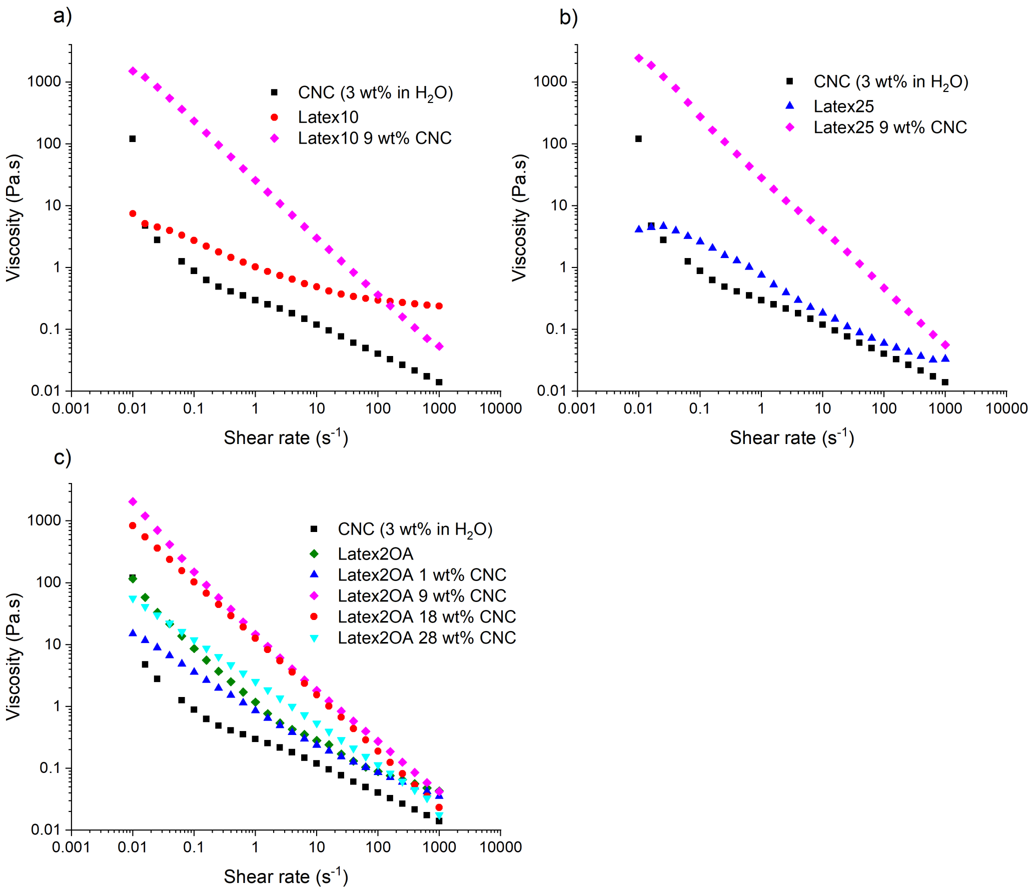

Figure S5. Flow sweep measurements of latex mixtures with CNC. a) Latex10. b) Latex25. c) Latex2OA.

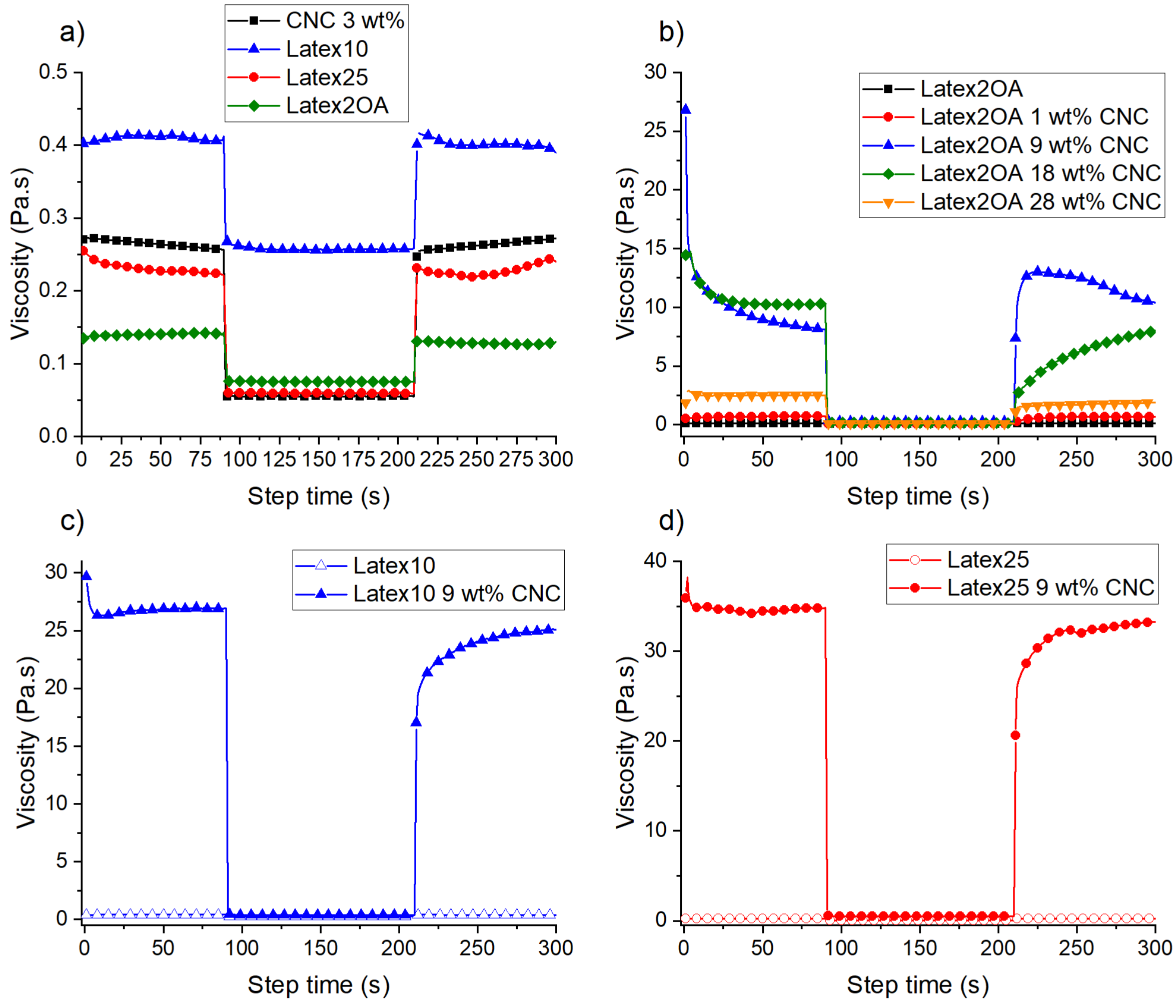

Figure S6. 3ITT curves alternating between low shear rate of 1 s^{-1} for 90 s, and a high shear rate of 100 s^{-1} for 120 s. a) The latexes and 3 wt% CNC dispersion. b) Latex2OA containing various amounts of CNC relative to solids. C) Latex10 containing 0 and 9 wt% CNC relative to solids. d) Latex25 containing 0 and 9 wt% CNC relative to solids.

Table S6. Viscosity recovery data from the 3ITT of latex mixtures with 3 wt% CNC dispersion.

Latex	CNC (wt%)	η_e (Pa.s) at		$\Delta\eta_e$ (Pa.s)	η (Pa.s) recovery after			$\eta_{(90-10)s}$ (Pa.s)
		1 s^{-1}	100 s^{-1}		1 s	10 s	90 s	
Latex10	9	26.9	0.4	26.5	17.0 (63%)	21.7 (81%)	25.1 (93%)	3.4
Latex25	9	34.8	0.5	34.3	20.6 (59%)	29.2 (84%)	33.2 (95%)	4.0
Latex2OA	1	0.7	0.1	0.6	0.3 (43%)	0.5 (71%)	0.7 (100%)	0.2
	9	8.2	0.3	7.9	7.4 (90%)	12.9 (157%)	10.4 (127%)	-2.5
	18	10.3	0.2	10.1	2.2 (21%)	3.7 (36%)	8.0 (78%)	4.3
	28	2.5	0.1	2.4	1.1 (44%)	1.6 (64%)	1.9 (76%)	0.3

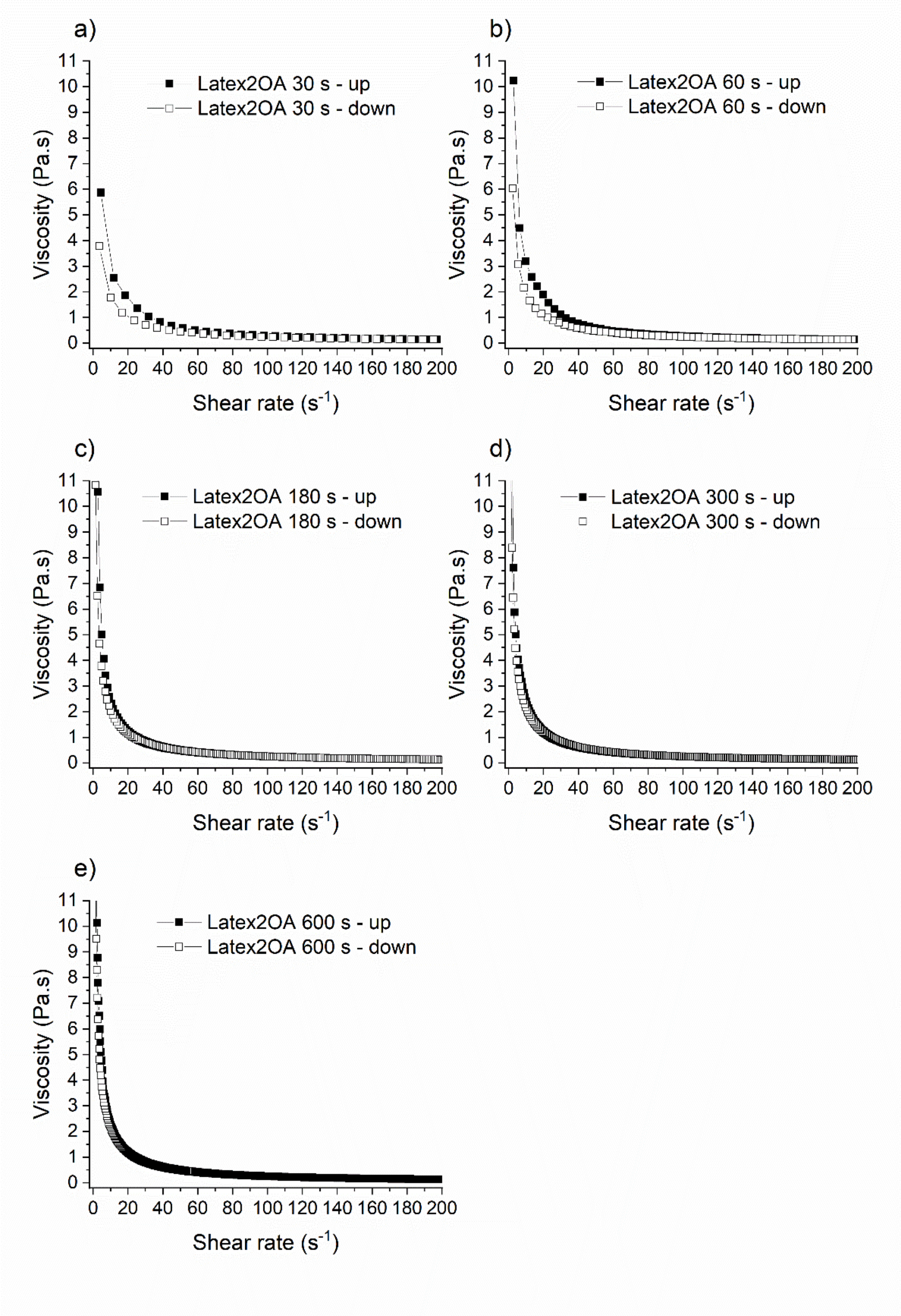

Figure S7. Rheology hysteresis curves of Latex2OA containing 9 wt% CNC, investigating the influence of the step time. a) 30s. b) 60 s. c) 180s. d) 300 s. e) 600 s.

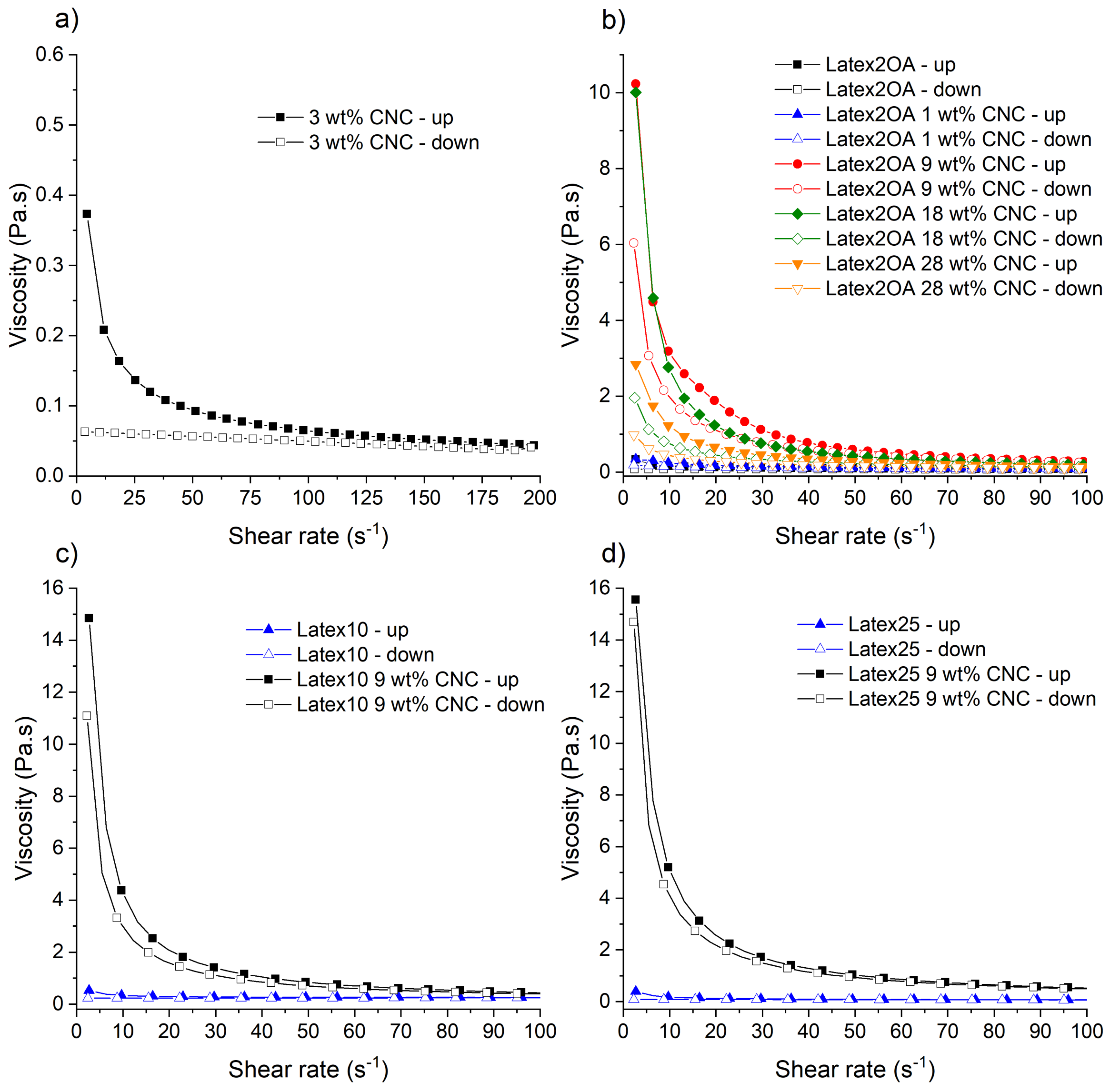

Figure S8. Rheology hysteresis loop curves with a step time of 60 s of a) 3 wt% CNC dispersion. b) Latex2OA mixed with various amount of CNC. c) Latex10 mixed with 9 wt% CNC. d) Latex25 mixed with 9 wt% CNC.

4. Supplementary tables and graphs

Table S7. DLS data of latexes directly after synthesis and after 11 months of storage at 4 °C.

Latex	Size initial	PDI initial	Size aged	PDI aged	Δ Size aged	Size freeze-thaw	PDI freeze-thaw	Δ Size freeze-thaw
	(nm)		(nm)		(nm)	(nm)		(nm)
Latex10	549	0.264	557	0.292	+8	664	0.403	+115
Latex25	187	0.010	182	0.061	-5	230	0.206	+43
Latex50	115	0.090	114	0.031	-1	109	0.021	-6
Latex2OA	136	0.142	132[a]	0.117[a]	-4	385	0.469	+249

[a] DLS spectrum was measured after 4 months of storage at 4 °C.

Table S8. Latex stability evaluation by freeze-thaw test, addition of 1 equivalent of salt solution and ethanol.

Latex	Freeze-thaw test	1.0 M NaCl	0.1 M $MgSO_4$	Ethanol
Latex10	+	+	+	+
Latex25	+	+	+	+
Latex50	+	+	+	+
Latex2OA	-	+	+	+

+ Macroscopically stable, no phase separation
- Macroscopically unstable, phase separation

Table S9. Surface active properties of the synthesized latexes.

Latex	Weight fraction POEGA in feed[a]	Unreacted surfactant (in supernatant)	Surface tension
	(%)	(%)	(mN/m)
Latex10	12.5	35.0	46.6
Latex25	12.0	38.2	44.8
Latex50	11.3	39.5	49.3
Latex2OA	11.4	45.5	49.1

[a] Relative to monomers.

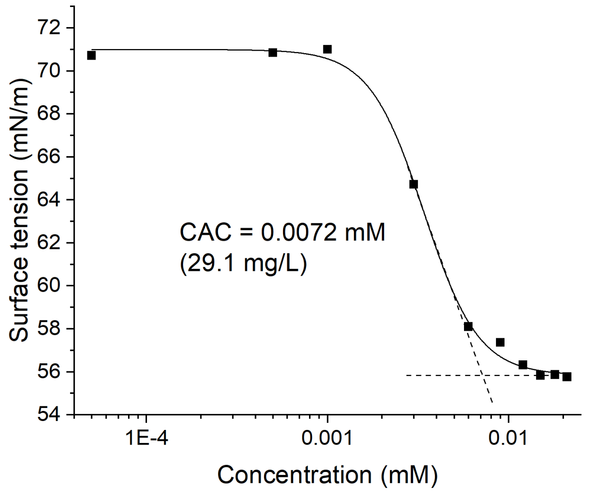

Figure S9. Surface tension as a function of the concentration for the POEGA macro-RAFT agent stabilizer in water.

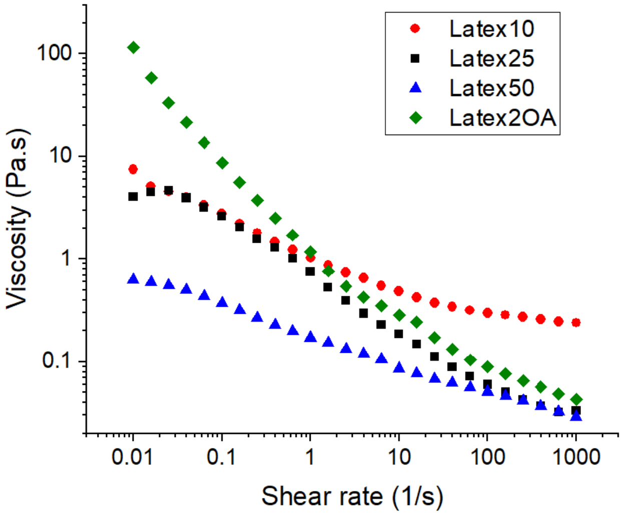

Figure S10. Rheology of the latexes in this work. Flow sweep measurement.

Table S10. Results of the freestanding films after 40 minutes UV irradiation from the synthesized latexes with various amount of CNC loading.

Code	CNC	E	σ_{max}	Strain at break	Water uptake[a]	Contact angle	Gel content THF[b]	Gel content H_2O[b]
	(wt%)	(MPa)	(MPa)	(%)	(%)	(°)	(wt%)	(wt%)
Latex10	0	749 ± 85	22.2 ± 1.6	12.4 ± 2.2	7.1 ± 0.9	71.3 ± 1.1	99.2	95.2
	1	641 ± 96	18.8 ± 1.6	9.5 ± 3.4	7.4 ± 0.5	60.3 ± 4.2	97.0	95.1
	9	1435 ± 62	23.7 ± 0.9	4.9 ± 0.9	9.5 ± 0.5	43.9 ± 3.1	97.8	94.9
Latex25	0	1052 ± 47	27.2 ± 2.1	5.8 ± 1.0	5.8 ± 0.2	83.7 ± 2.0	98.2	94.9
	1	833 ± 101	27.6 ± 3.5	10.4 ± 2.2	5.8 ± 0.2	86.3 ± 1.6	97.1	95.4
	9	1339 ± 220	31.6 ± 1.0	8.2 ± 1.4	7.5 ± 0.3	86.7 ± 3.8	98.4	95.0
Latex50	0	1248 ± 117	32.4 ± 2.0	5.4 ± 1.0	4.1 ± 1.2	94.2 ± 2.8	99.7	95.6
Latex-2OA	0	63 ± 16	4.5 ± 0.5	13.0 ± 1.8	4.5 ± 0.5	94.7 ± 0.8	99.8	95.4
	1	86 ± 13	5.3 ± 0.3	12.3 ± 0.9	3.6 ± 0.4	99.1 ± 1.8	97.1	95.4
	9	277 ± 16	9.6 ± 1.1	13.1 ± 2.6	6.5 ± 1.0	91.6 ± 2.4	97.7	95.5
	18	702 ± 42	20.5 ± 1.5	16.0 ± 3.2	6.4 ± 0.4	104.6 ± 1.5	97.7	95.5
	28	1386 ± 170	28.0 ± 2.9	8.0 ± 1.6	7.7 ± 0.3	104.1 ± 1.4	98.1	95.7
	40	3075 ± 272	50.8 ± 3.9	5.4 ± 1.2	10.3 ± 0.7	95.0 ± 4.5	99.0	93.6
	60	5018 ± 469	66.9 ± 0.8	2.5 ± 0.1	30.6 ± 0.2	77.4 ± 2.9	99.8	94.1
	80	8330 ± 798	79.4 ± 19.6	1.3 ± 0.4	88.1 ± 4.4	91.4 ± 5.0	99.8	93.9

[a] After immersion in H_2O for 48h. [b] After 24 hours Soxhlet extraction. The errors reported are the standard deviation between repeated measurements.

Table S11. Tensile properties of freestanding films.

Code	Curing time	E	σ_{max}	Strain at break
	(min)	(MPa)	(MPa)	(%)
Latex10	20	370 ± 49	12.5 ± 0.7	11.0 ± 1.6
	10	136 ± 8.0	9.0 ± 1.1	34.7 ± 4.4
	5	24 ± 7.7	4.2 ± 1.1	60.3 ± 7.6
	0	1.8 ± 0.7	0.6 ± 0.1	80 ± 32.3
Latex25	60	965 ± 65	25.6 ± 1.5	5.7 ± 0.9
[a]	40	721 ± 39	21.4 ± 0.9	11.3 ± 3.5

[a] Conditions were the same as Latex25 native film but the film thickness was about three times thicker (0.61 ± 0.06 mm).

Table S12. Properties of the reference latex containing 10 mol% 4CPA.

Latex	Molar feed ratio			Time	Monomer conversion (%)			T_g	Gel content	Size	PDI	Solids[a]
	4CPA	THFA	IBOA	(min)	4CPA	THFA	IBOA	(°C)	(wt%)	(nm)		(%)
RefLatex	10	80	10	120	99	97	99	-8.8	84	184	0.108	30

[a] Theoretical amount.

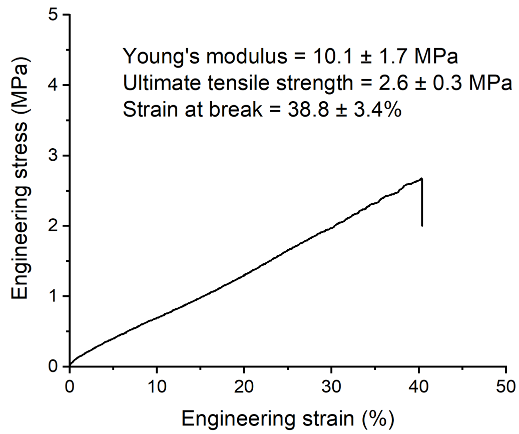

Figure S11. Tensile graph of a UV cured film from the reference latex containing 10 mol% 4CPA. The film, with a thickness of about 0.2 mm was UV cured for 20 minutes on each side.

Table S13. Overview of the TGA data of the unmodified and cross-linked freestanding films.

Latex	UV curing time	$T_{5\%}$	Residue
	(min)	(°C)	(%)
Latex10	0	256	5.3
	40	248	8.7
Latex25	0	251	6.2
	40	239	7.8
Latex50	0	258	6.0
	40	240	7.9
Latex2OA	0	259	6.5
	40	225	8.2

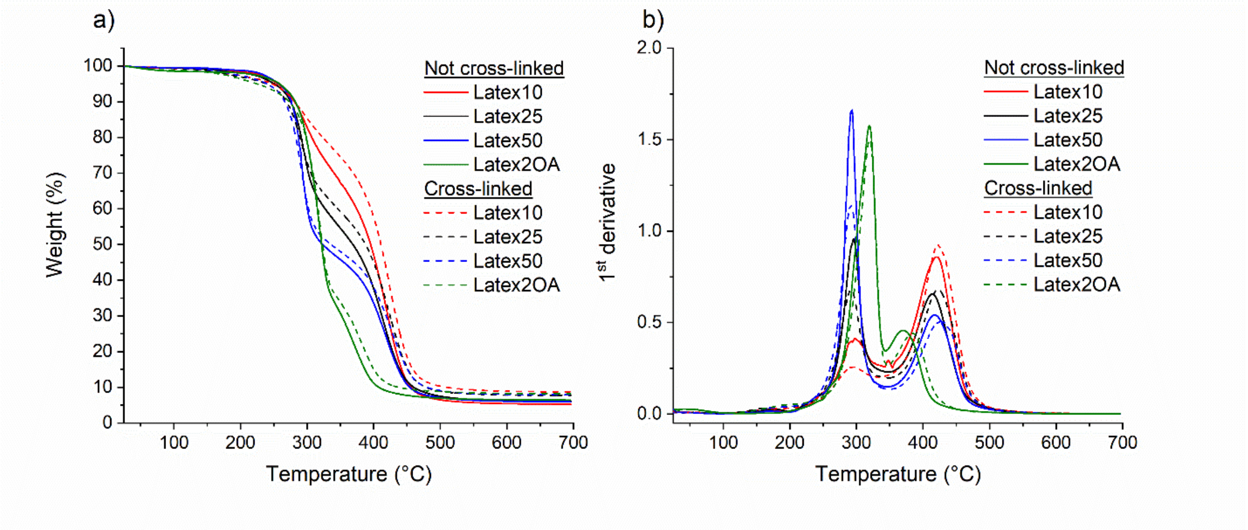

Figure S12. TGA results of unmodified and cross-linked freestanding films. a) Weight loss graph. b) 1st derivative as a function of the temperature.

Table S14. Literature overview of the Cobb water absorption values after 60 seconds (Cobb60) of various reference coatings on paper and paperboard.

**Material**	**Substrate**	**Substrate weight**	**Uncoated Substrate Cobb60**	**Dry coating weight**	**Coated Cobb60**
		**(g/m^2)**	**(g/m^2)**	**(g/m^2)**	**(g/m^2)**
Material in this study	Paper	78	70.8	3.3	1.6
Material in this study	Paper	78	70.8	27.6	0.3
polyurethane/poly(n-butyl acrylate-styrene) hybrid emulsions[5]	Paper	75	22.7	N/A	2.7
Starch based coating[6]	Paperboard	261	39	10	20
Chitosan-Zein[7]	Paper	35	29.3	23.6	4.9
Polylactic acid*[8]	Paper	110	25.6	9	3.2
Zein-PDMS[9]	Paperboard	144	36	17.4	6.5
Acrylic latex/styrene-butadiene latex formulation**[10]	Paperboard	303	N/A	0.78	13-30

* 30s Cobb test

** 120s Cobb test

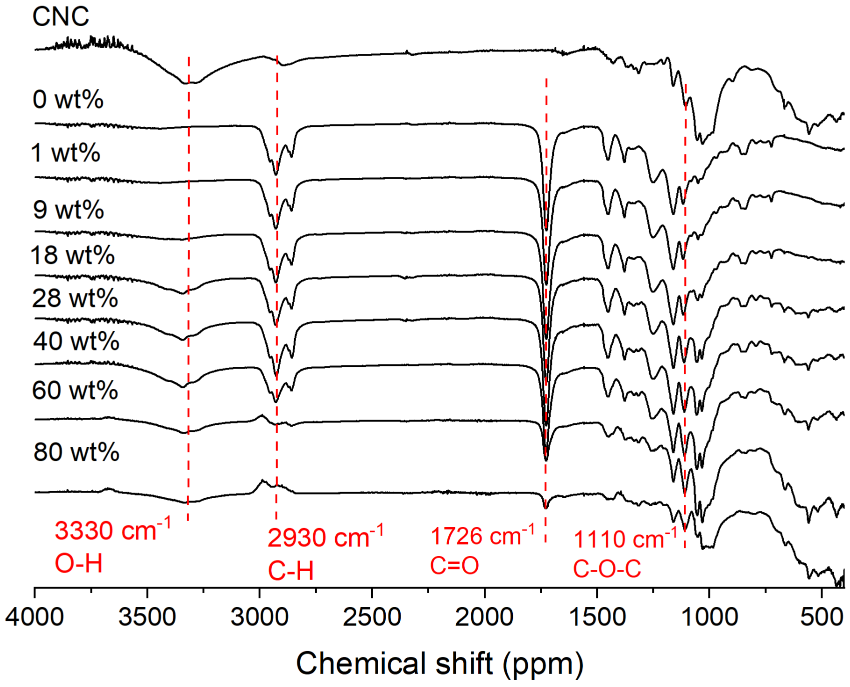

Figure S13. Overlay of FTIR spectra of the CNC loaded films.

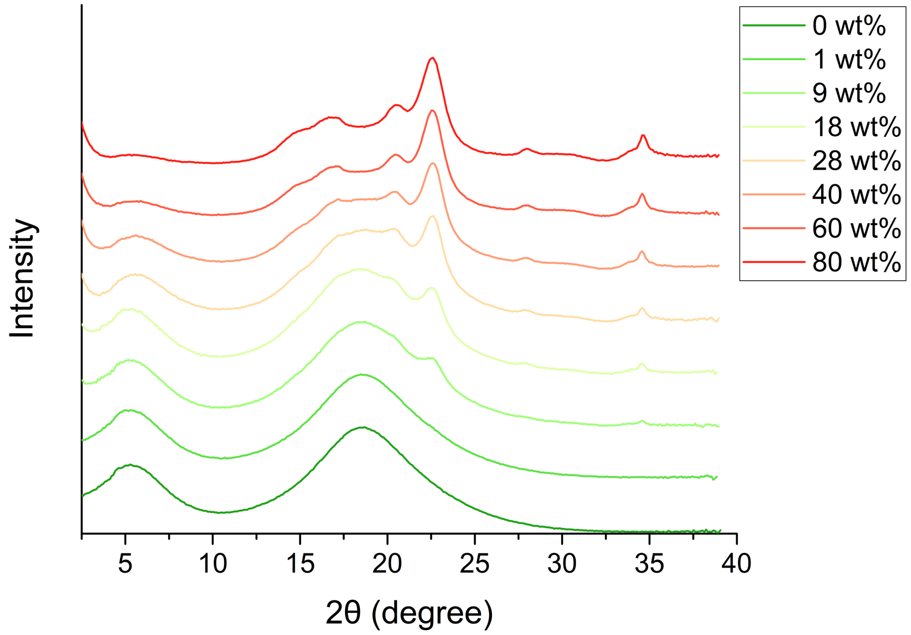

Figure S14. Overlay of 1D WAXD curves of the CNC loaded films.

REFERENCES

1. Jaacks, V., A Novel Method of Determination of Reactivity Ratios in Binary and Ternary Copolymerizations. *Die Makromolekulare Chemie: Macromolecular Chemistry and Physics* **1972,** *161* (1), 161-172.
2. Stouten, J.; Vanpoucke, D. E.; Van Assche, G.; Bernaerts, K. V., UV-Curable Biobased Polyacrylates Based on a Multifunctional Monomer Derived from Furfural. *Macromolecules* **2020,** *53* (4), 1388-1404.
3. Vancoillie, G.; Frank, D.; Hoogenboom, R., Thermoresponsive Poly (oligo ethylene glycol acrylates). *Progress in Polymer Science* **2014,** *39* (6), 1074-1095.
4. Lu, M.; Song, C.; Wan, B., Influence of Prepolymer Molecular Weight on the Rheology and Kinetics of HEUR-Thickened Latex Suspensions. *Progress in Organic Coatings* **2021,** *156*, 106223.
5. Guo, Y.-h.; Li, S.-c.; Wang, G.-s.; Ma, W.; Huang, Z., Waterborne Polyurethane/Poly (n-Butyl Acrylate-Styrene) Hybrid Emulsions: Particle Formation, Film Properties, and Application. *Progress in Organic Coatings* **2012,** *74* (1), 248-256.
6. Chi, K.; Wang, H.; Catchmark, J. M., Sustainable Starch-Based Barrier Coatings for Packaging Applications. *Food Hydrocolloids* **2020,** *103*, 105696.
7. Kansal, D.; Hamdani, S. S.; Ping, R.; Sirinakbumrung, N.; Rabnawaz, M., Food-Safe Chitosan–Zein Dual-Layer Coating for Water-and Oil-Repellent Paper Substrates. *ACS Sustainable Chemistry & Engineering* **2020,** *8* (17), 6887-6897.
8. Sundar, N.; Kumar, A.; Pavithra, A.; Ghosh, S., Studies on Semi-Crystalline Poly Lactic Acid (PLA) as a Hydrophobic Coating Material on Kraft Paper for Imparting Barrier Properties in Coated Abrasive Applications. *Progress in Organic Coatings* **2020,** *145*, 105682.
9. Hamdani, S. S.; Li, Z.; Rabnawaz, M.; Kamdem, D. P.; Khan, B. A., Chitosan–Graft–Poly (Dimethylsiloxane)/Zein Coatings for the Fabrication of Environmentally Friendly Oil-and Water-Resistant Paper. *ACS Sustainable Chemistry & Engineering* **2020,** *8* (13), 5147-5155.
10. Yongping, Z.; Gordon, V.; Lucci, S.; Schottland, P. Grease, Oil, and Water Resistant Coating Compositions. US11242461B2, 2022.
